# Investigation of F-BAR domain PACSIN proteins uncovers membrane tubulation function in cilia assembly and transport

**DOI:** 10.1038/s41467-018-08192-9

**Published:** 2019-01-25

**Authors:** Christine Insinna, Quanlong Lu, Isabella Teixeira, Adam Harned, Elizabeth M. Semler, Jim Stauffer, Valentin Magidson, Ajit Tiwari, Anne K. Kenworthy, Kedar Narayan, Christopher J. Westlake

**Affiliations:** 1 Laboratory of Cellular and Developmental Signaling, Center for Cancer Research, National Cancer Institute, National Institutes of Health, Frederick, MD 21702 USA; 2 Center for Molecular Microscopy, Center for Cancer Research, National Cancer Institute, National Institutes of Health, Frederick, MD 21701 USA; 3 Cancer Research Technology Program, Frederick National Laboratory for Cancer Research, Frederick, MD 21702 USA; 4 Department of Molecular Physiology and Biophysics, Vanderbilt University School of Medicine, Nashville, TN 37232 USA

## Abstract

The intracellular ciliogenesis pathway requires membrane trafficking, fusion, and reorganization. Here, we demonstrate in human cells and zebrafish that the F-BAR domain containing proteins PACSIN1 and -2 play an essential role in ciliogenesis, similar to their binding partner and membrane reorganizer EHD1. In mature cilia, PACSINs and EHDs are dynamically localized to the ciliary pocket membrane (CPM) and transported away from this structure on membrane tubules along with proteins that exit the cilium. PACSINs function early in ciliogenesis at the ciliary vesicle (CV) stage to promote mother centriole to basal body transition. Remarkably, we show that PACSIN1 and EHD1 assemble membrane tubules from the developing intracellular cilium that attach to the plasma membrane, creating an extracellular membrane channel (EMC) to the outside of the cell.

## Abstract (web summary)

PACSIN proteins are known to mediate membrane tubulation. Here the authors show that PACSIN - positive membranous tubules extend from the ciliary pocket membrane and during ciliogenesis from the ciliary vesicle to the plasma membrane, called extracellular membrane channels (EMCs).

## Introduction

Defects in cilia are linked to human genetic diseases called ciliopathies, and cancer^1,2^. Ciliogenesis is a cell cycle-regulated process, with cilia growing in interphase or G_0_, and resorbing prior to mitosis. Ciliogenesis occurs via two distinct processes, the extracellular and intracellular pathways^3–6^. In the extracellular pathway, the mother centriole (MC) directly docks with the plasma membrane (PM) prior to axonemal growth, whereas in the intracellular pathway, the cilium begins to develop in the cytoplasm and fuses with the PM through an unknown mechanism. Before the assembly of the microtubule-based axoneme, distal appendages proteins of the MC mediate association with cellular membranes to promote removal of the CP110/CEP97 cap from the MC distal end^7^. During intracellular ciliogenesis, preciliary membrane vesicles traffic to the MC, dock to the distal appendages (called distal appendage vesicles or DAVs) and fuse into a larger ciliary vesicle (CV)^8^. CV assembly promotes the removal of the CP110/CEP97 complex and leads to the recruitment of intraflagellar transport (IFT) and transition zone (TZ) proteins followed by axonemal growth^8^. Abnormal progression through the intracellular pathway has been observed in ciliopathy patient fibroblasts and human astrocytoma/glioblastoma cell lines^9,10^.

Membrane trafficking regulators such as the small GTPases Rab and Arl family members are important for intracellular ciliogenesis^11–18^. The Rab11–Rab8 cascade plays a critical role in early ciliary assembly inside the cell^11,13^. Rab11 transports preciliary membrane vesicles and ciliogenic proteins to the MC, including the Rab8 guanine nucleotide exchange factor Rabin8, while Rab8 promotes ciliary membrane growth from the CV. Other trafficking regulators, such as components of the exocyst and TRAPPI/II complexes and SNARE membrane fusion proteins also function in intracellular ciliogenesis^8,13,19^. Recently, we demonstrated that the membrane trafficking regulators Eps15 homology domain (EHD)-family of proteins EHD1 and -3 serve critical roles for CV assembly, possibly through DAV reshaping and/or recruitment of the membrane fusion protein SNAP29^8^. A direct role for EHDs in membrane reorganization processes is not clear, as these proteins require orchestration with additional factors to assist in shaping and remodeling lipid bilayers. Such functions can be achieved by the F-BAR domain-containing protein kinase C and casein kinase II interacting protein (PACSIN) family. PACSINs, also referred to as Syndapins, form homo- and hetero-dimers that confer the ability to sense membrane curvature and tubulate lipid bilayers through high-ordered lattice organization formed by tip-to–tip interactions^20–22^. The mammalian isoforms PACSIN 1 and -2, but not PACSIN3, interact with EHD1 and -3 through their NPF motifs, while the C-terminal SH3 domains associate with proteins involved in various functions including endocytosis, endosomal vesicle trafficking, and cytoskeletal remodeling^20,23–28^. In zebrafish, loss of Pacsin1b leads to lateral line ciliary defects and developmental abnormalities typically associated with ciliogenic impairment^29^.

Here, we show that PACSIN1 and -2 have cell/tissue-specific functions at the CV stage in ciliogenesis. These proteins dynamically localize to membrane tubules forming off the emerging CV/short intracellular cilium and the ciliary pocket membrane (CPM) in the mature cilium of cultured cells and zebrafish embryos. Remarkably, we show that PACSIN/EHD-positive membrane tubules connect the developing intracellular cilium with the cell surface, creating a route to the outside of the cell. Functional requirements for PACSIN1, EHD1, and microtubules in the establishment of an extracellular membrane channel (EMC) are demonstrated. Our findings define the role of membrane shaping proteins in ciliogenesis and uncover the mechanism by which the intracellular cilium fuses with the PM.

## Results

### PACSIN 1 and -2 are required for ciliogenesis

**Fig. 1:**
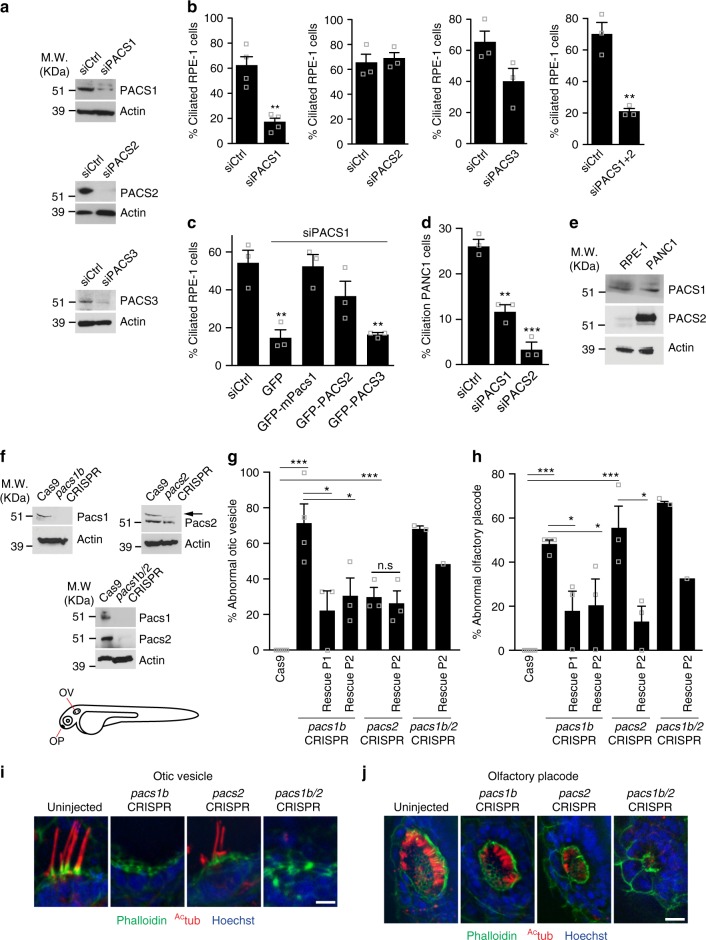
PACSIN1 and -2 are required for ciliogenesis. **a** Western analysis of PACSINs depletion in RPE-1 cells treated with siCtrl or siPACSINs (sequences in Supplementary Table 1). **b** Quantification of ciliated cells treated as in **a** (*n* = 4 independent experiments, siCtrl = 801, siPACS1 = 543 cells in total; *n* = 3, Ctrl = 215, siPACS2 = 128 cells; *n* = 3, siCtrl = 107, siPACS3 = 85 cells; *n* = 3, siCtrl = 106, siPACS1+2 = 75 cells). Images are in Supplementary Figure 1a. **c** Quantification of ciliation in RPE-1 cells treated with siPACSIN1 for 6 h, rescued with indicated constructs and starved at 48 hpf (*n* = 3, siCtrl = 108, GFP = 71, GFP-mPacs1 = 39, GFP-PACS2 = 75, GFP-PACS3 = 55 cells). **d** Quantification of ciliation in PANC1 cells treated as in **b** (*n* = 3, siCtrl = 273, siPACS1 = 194, siPACS2 = 175 cells). **e** Western analysis of PACSINs expression in RPE-1 and PANC1 cells. **f** Western analysis of Pacsins expression in 3 days post fertilization (dpf) CRISPR mutants (gRNA sequences shown in Supplementary Table 1). Arrow indicates Pacsin2 band. Schematic of organs of interest (Otic vesicle: OV, and Olfactory placode: OP). **g** Quantification of abnormal OV from embryos as in **f** and rescued with human PACSIN RNAs. Cas9, 38 OVs, *n* = 5; *pacs1b*CRISPR, 37 OVs, *n* = 4; *pacs1b*CRISPR + hPACSIN1, 20 OVs, *n* = 3; *pacs1b*CRISPR + hPACSIN2, 16 OVs, *n* = 3; *pacs2*CRISPR, 23 OVs, *n* = 3; *pacs2*CRISPR + *hPACSIN2*, 20 OVs, *n* = 3; *pacs1b/2*CRISPR, 15 OVs, *n* = 2; *pacs1b/2*CRISPR + hPACSIN2, 5 OVs, *n* = 1. Images are shown in (**i**) (scale bar: 5 μm). **h** Quantification of abnormal OP in embryos injected as in **g**. Cas9, 37 OPs, *n* = 5; *pacs1b*CRISPR, 25 OPs, *n* = 4; *pacs1b*CRISPR + hPACSIN1, 17 OPs, *n* = 3; *pacs1b*CRISPR + hPACSIN2, 14 OPs, *n* = 3; *pacs2*CRISPR, 18 OPs, *n* = 3; *pacs2*CRISPR + *hPACSIN2*, 19 OPs, *n* = 3; *pacs1b/2*CRISPR, 15 OPs, *n* = 2; *pacs1b/2*CRISPR + hPACSIN2, 6 OPs, *n* = 1. Images are shown in **j** (scale bar: 10 μm). Images in **i** and **j** are maximum intensity projections of deconvolved z-stacks. Means ± SEM. Two-tailed *t*-test analyses as compared with siCtrl in **b** and **d** or as indicated in figure. *P < 0.05, **P < 0.001, non significant (n.s)

We investigated the ciliogenic function of the EHD1 and -3 interacting protein PACSIN family to further elucidate membrane reorganization processes at the MC^8^. RNAi-mediated knockdown of PACSIN1, but not PACSIN2 and -3, resulted in ciliogenesis defects in hTERT-RPE1 (RPE-1) cells (Fig. 1a, b, Supplementary Figure 1a). Ciliation was rescued by siRNA-resistant murine GFP-Pacsin1 and GFP-PACSIN2, but not GFP-PACSIN3 or GFP (Fig. 1c). This validated the specificity of the knockdown, and suggested that PACSIN1 and PACSIN2 have ciliogenic functions, whereas PACSIN3, which lacks the EHD binding NPF motif^23^, is dispensable for this process. Depletion of both PACSIN1 and -2 did not enhance ciliation defects, suggesting that PACSIN1 is sufficient for ciliogenesis in RPE-1 cells (Fig. 1b). In contrast, PACSIN2 depletion had a stronger effect in PANC1 and normal pancreatic hTERT-HPNE (HPNE) cells, while both PACSINs were important in neonatal foreskin-derived fibroblasts (NeoHFF) cells (Fig. 1d, Supplementary Figure 1b). These cell-specific ciliogenic functions correlated with PACSIN expression levels (Fig. 1e, Supplementary Figure 1c). PACSIN2 depletion in murine Inner Medullary Collecting Duct (mIMCD3) cells (Supplementary Figure 1d), a line thought to use the extracellular ciliogenesis pathway^6^ and expressing low levels of PACSIN1 (Supplementary Figure 1e), did not affect ciliogenesis (Supplementary Figure 1f), consistent with a previous report^30^.

To further investigate PACSIN1 and -2 ciliogenic requirements, we generated *pacsin1b* and *pacsin2* zebrafish mutants using CRISPR/Cas9. Suppression of Pacsin1 and Pacsin2 expression was confirmed by western blotting (Fig. 1f). As in mammalian cells, *pacsin* mutants exhibited ciliogenesis defects (Fig. 1g–j). Interestingly, Pacsin1b was required for otic vesicle (OV) ciliation and hPACSIN2 did not rescue the mild ciliogenesis defects of the *pacsin2* CRISPR, suggesting a minor contribution of Pacsin2 in OV ciliogenesis (Fig. 1g, i), while both Pacsin proteins were important for olfactory placode (OP) cilia development (Fig. 1h, j). As in human cell lines, tissue-specific requirements for ciliogenesis correlated with Pacsin expression (Supplementary Figure 2a, b). In support of this, stronger effects were observed in OP of *pacsin2* mutants and synergistic effects were observed in double mutant embryos (Fig. 1h, j), which also displayed morphological phenotypes consistent with ciliogenic impairment (body curvature, smaller eyes, and hydrocephalus, Supplementary Figure 2c). Human PACSIN1 and/or PACSIN2 RNA successfully rescued ciliogenesis defects induced by *pacsin* single and double mutants in both organs, validating the CRISPR effects are specific (Fig. 1g, h). Impaired ciliation was also observed in the tail of double mutant animals (Supplementary Figure 2d). Taken together, our results suggest redundant and tissue-specific functions for PACSINS in ciliogenesis of human and zebrafish cells.

### PACSIN and EHD proteins display dynamic CPM trafficking with EHD1

**Fig. 2:**
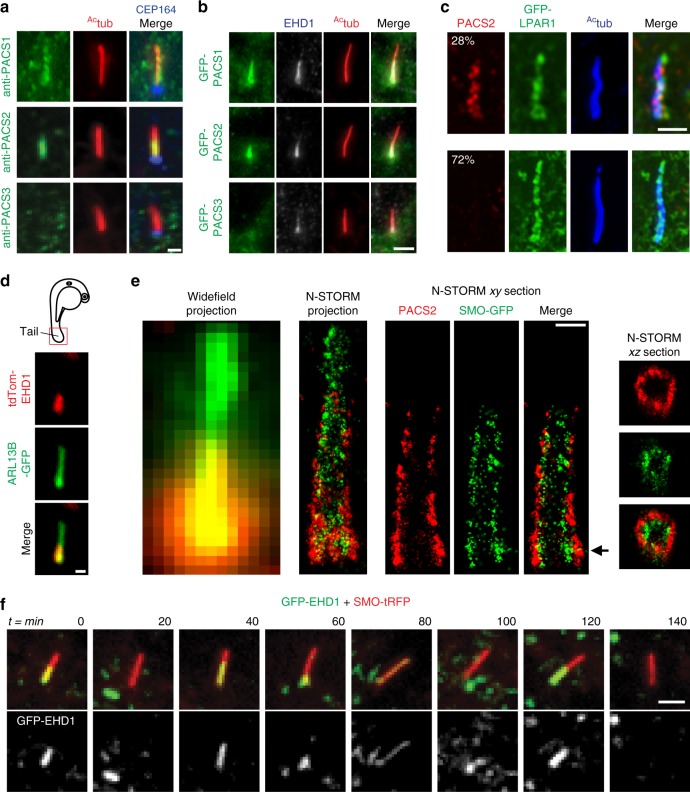
PACSIN and EHD proteins display dynamic CPM trafficking. **a** Representative images of RPE-1 cells serum starved for 24 h and stained with antibodies for PACSINs, ^Ac^tub, and CEP164. PACSIN2 antibody showed lower levels of background staining in RPE-1 cells as compared to the PACSIN1 antibody. **b** Representative images of GFP-PACSINs expressed in RPE-1 cells as in **a** and stained with antibodies for EHD1 and ^Ac^tub. Note that GFP-PACSIN1 and GFP-PACSIN2 always co-localized with EHD1 (GFP-PACSIN1 = 85, GFP-PACSIN2 = 90 cells). SDC images in **a** and **b** are maximum intensity z-projections. Scale bar: 2 μm. **c** N-SIM images of cilia from cells transiently transfected with GFP-LPAR1, serum starved for 24 h, and stained with ^Ac^tub and PACSIN2 antibodies. Images are single *xy* planes. Scale bar: 2 μm. **d** Representative image of tdTom-EHD1 and the ciliary membrane marker ARL13B-GFP expressed in a cilium from the tail region (red box in schematic; top panel) of a 24 hpf embryo. SDC images are maximum intensity z-projections. Scale bar: 1 μm. **e** Representative images of epifluorescence (left panel) and N-STORM projections (middle left panel) from 24 h serum-starved SMO-GFP RPE-1 cells stained with PACSIN2 antibody as described in Methods. Images of a single *xy* plane bisecting the cilium (middle right panels). Orthogonal views of N-STORM images corresponding to the black arrow (right panels). Scale bar: 300 nm. **f** GFP-EHD1 + SMO-tRFP cells were serum starved for 24 h and imaged live every 20 min. Images are single *xy* planes (30 ciliated cells). Scale bar: 2 μm

Because human PACSIN1 and -2 are important for ciliogenesis, we examined if these proteins are localized to cilia. Endogenous PACSIN1 and -2, but not PACSIN3, were detected in the proximal ciliary region of RPE-1 cells (Fig. 2a), a localization previously reported for Pacsin1 in zebrafish ZF4 cells^29^. This localization was also observed in NeoHFF, HPNE, and PANC1 cells (Supplementary Figure 3a). PACSIN2 was found associated with 28% of RPE-1 cells cilia (125 cells), and both endogenous and exogenous PACSINs always co-localized with EHD1 proteins (Fig. 2b, c, Supplementary Figure 3b, c), consistent with EHD1 and PACSINs forming complexes on intracellular membranes^23^. Similarly, tdTom-EHD1 was detected in the proximal ciliary region in 12% of cilia (164 cilia from 5 independent fish) from cells in the tails of 24 hpf zebrafish embryos (Fig. 2d). Previously, we demonstrated that EHD1 localizes to the CPM^8^. To confirm PACSIN localization, we performed one- and two-color stochastic optical reconstruction microscopy (N-STORM), which can resolve structures <50 nm in *xy*, in RPE-1 cells expressing the ciliary membrane marker Smoothened (SMO) tagged with GFP and PACSIN2 antibodies (Fig. 2e). Consistent with a recent report^31^, N-STORM imaged SMO-GFP had a ciliary diameter of 232 ± 21 nm (±SD, 6 cells). In contrast, PACSIN2 was detected outside of the SMO-GFP signal with a diameter of 347 ± 16 nm (±SD, 11 cells), indicating that PACSINs, like EHD1, are present on the CPM.

We next considered why PACSIN and EHD proteins only localized to the CPM in some cells. Using the non-ciliary PM integral membrane protein lysophosphatidic acid receptor (LPAR1) fused to GFP, a PM and CPM marker (Fig. 2c, Supplementary Figure 3d, e), we confirmed that as reported previously^6,32^, ~95% of RPE-1 cells (74 cells) have a CPM (Fig. 2c), suggesting that the lower frequency of PACSIN/EHD proteins at the CPM may be attributed to specific or dynamic trafficking of these proteins. To test these theories, we used live-cell imaging. GFP-PACSIN1 and -2 fusions were not suitable for these experiments due to their weak CPM localization (Fig. 2b) and rapid photobleaching. Instead, we used a previously described^8^ cell line stably expressing ciliary SMO fused to Tag-RFP (tRFP) and GFP-EHD1, which was expressed ~3-fold higher than endogenous EHD1 (Supplementary Figure 3f). Remarkably, GFP-EHD1 trafficking in and out of the CPM could be observed within minutes (Fig. 2f). Together, these results indicate that EHD and PACSIN proteins have dynamic CPM localization.

### PACSIN and EHD proteins form membrane tubules from the CPM

**Fig. 3:**
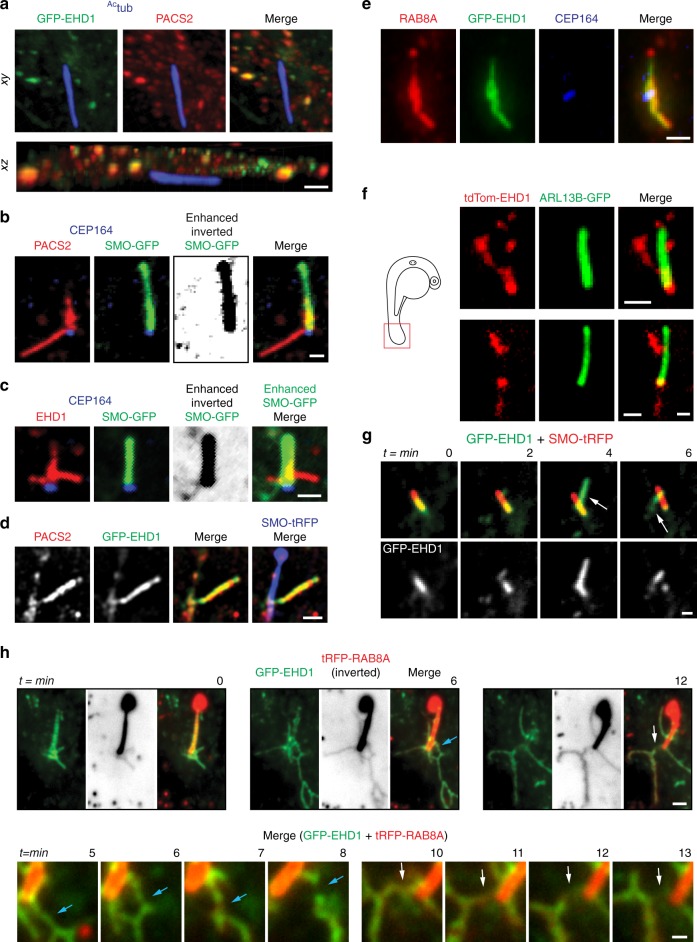
PACSIN and EHD proteins accumulate on CPM tubules that contain RAB8A. **a** Representative 3D volume view images (generated by SlideBook) of GFP-EHD1 cells serum starved for 24 h and stained with antibodies to PACSIN2 and ^Ac^tub. The z-stack was captured using a SDC microscope and a CMOS camera. **b**, **c** SMO-GFP cells imaged as in **a** and stained with antibodies to PACSIN2 or EHD1, CEP164, and anti-GFP. Contrast enhanced and inverted image in the middle right panel demonstrate the absence of SMO-GFP in PACSIN2/EHD1 tubules. **d** Representative N-SIM maximum intensity projection images of CPM-associated membrane tubules in ciliated GFP-EHD1 (green) + SMO-tRFP (pseudo-colored blue) cells stained with PACSIN2 antibody (pseudo-colored red). **e** Epifluorescence projected z-stack images of a GFP-EHD1-positive CPM-tubule in ciliated cells stained with RAB8A and CEP164 antibodies highlighting the presence of endogenous RAB8A in both the ciliary membrane and the CPM tubules (7 cells). **f** Representative image of CPM-associated membrane tubules in tail cilia of 24 hpf zebrafish embryos expressing tdTom-EHD1 and ARL13B-GFP imaged by SDC microscope with a CMOS camera. Tail region is represented by red box in schematic of zebrafish embryo on the left. **g** GFP-EHD1 + SMO-tRFP cells were starved for 24 h and imaged live every 2 min (16 ciliated cells). Arrows mark dynamic tubules associated with the CPM over time. **h** GFP-EHD1 cells transiently transfected with tRFP-RAB8A, starved for 24 h, and imaged live using TIRF-M (upper panels). tRFP-RAB8A signal is shown inverted (middle images) and in red (merged right images). Arrows indicate breaks in membrane tubules. Scale bar: 1 μm. Enlarged regions (lower panels) from upper images showing membrane tubule breaks with additional time-lapse images added (12 cilia). Scale bar: 500 nm. Images in (**g**) and (**h**) are single *xy* planes. Scale bars: 1 μm for all images unless specified

**Fig. 4:**
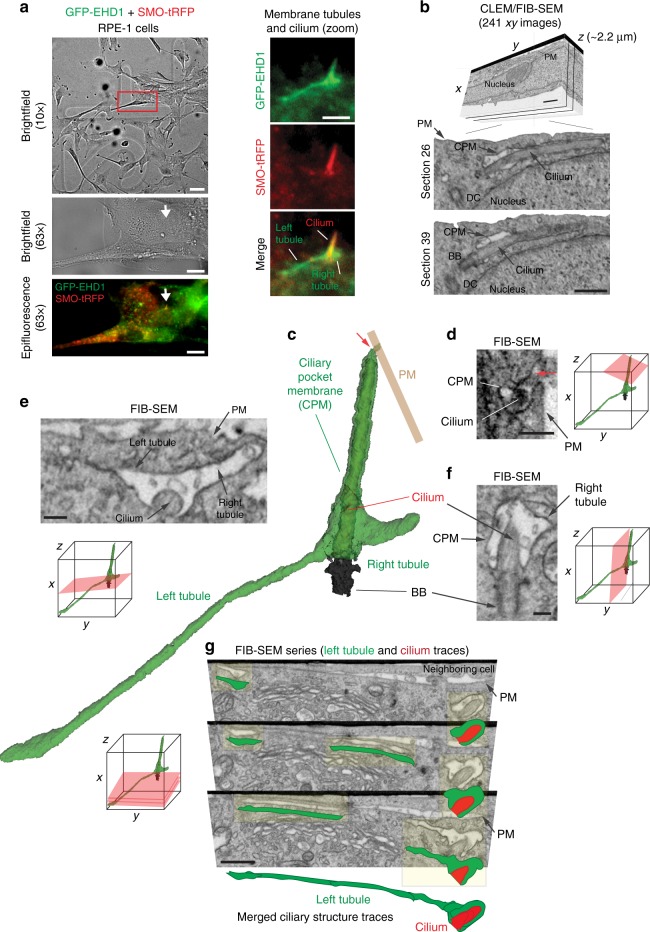
Three-dimensional FIB-SEM analysis shows membrane tubules connected to the CPM. **a** Brightfield and epifluorescence images of serum starved GFP-EHD1 + SMO-tRFP cells with membrane tubules associated with the cilium. Left panels show brightfield and fluorescence images at 10× and 63× used to identify the position of the ciliated cell on an alphanumerical grid for CLEM/FIB-SEM analysis (red box in top panel). Right panels show a 63× zoom of the cilium and associated membrane tubules (white arrow in the left panels). Scale bars: left top panel: 50 μm, left middle and bottom panels: 10 μm, right panels: 2 μm. **b** FIB-SEM image stack of the cell in **a**. Top panel shows a volume view of 241 *xy* plane FIB-SEM images (1102 × 472 pixels; 9 nm pixel size) with a cell depth (z) of ~2.2 μm (top panel). Middle and bottom panels show cropped FIB-SEM images of xy sections 26 and 39 with the cilium, CPM, basal body (BB), and daughter centriole (DC). Scale bars: 1 μm. The raw FIB-SEM image stack for the ciliary structures is available in Supplemental Movie 2. **c** 3D segmentation analysis of the FIB-SEM images in **b** using 3DSlicer software. Cilium (red), BB (black), CPM connected to the PM (red arrow) and the left and right CPM tubules (green). **d**–**f** FIB-SEM images showing the connection of the CPM with the PM (**d**, red arrow), transverse (**e**), and longitudinal (**f**) sections of the cilium from **b**. **e**, **f** show a shorter tubule (right tubule) and a longer tubule (left tubule), both attached to the CPM. Scale bars: 500 nm (**d**), 200 nm (**e**, **f**). **g** Representative FIB-SEM images (top three panels) from **b** showing the continuous left tubule connected to the CPM. Traces for the cilium (red) and the left tubule (green) are shown offset from FIB-SEM structures (yellow highlights) and merged in the bottom panel. Scale bar: 1 μm. **d**–**g** Orientation (red planar sheets) of the FIB-SEM images is indicated in the *xyz* model. **b**, **d**–**g** Images were generated with IMOD. CPM membrane tubules were observed by CLEM/FIB-SEM (2 cells)

We further investigated the dynamics of PACSINs and EHD1 proteins trafficking at the CPM. Consistent with previous reports^23^, PACSIN2 co-localizes with EHD1 on intracellular vesicles and at the cell surface (Fig. 3a), both being possible transport routes to the CPM^33,34^. Strikingly, we found membrane tubule-like structures at the CPM associated with PACSIN2 (8%, 249 cilia), or EHD1 (7%, 31 cilia) in RPE-1 cells (Fig. 3b, c, Supplementary Figure 4a, b), and PACSIN1 in both RPE-1 and NeoHFF cells (Supplementary Figure 4c, d). GFP-EHD1 tubule-like CPM structures (Fig. 3d, e) were found in 11% (200 cilia) of ciliated RPE-1 cells, and always had co-localized PACSIN1 and PACSIN2 (Fig. 3d, Supplementary Figure 4c). Moreover, tdTom-EHD1 tubule-like CPM structures could also be observed in ~2.5% of ARL13B-GFP-positive cilia (164 cilia) from cells in the zebrafish tail region (Fig. 3f). Next, we performed live-cell microscopy on RPE-1 cells expressing GFP-EHD1 and ciliary-tRFP markers. Remarkably, loss of GFP-EHD1 from the CPM in RPE-1 cells correlated with the generation of tubules (Fig. 3g, h, Supplementary Movie 1), suggesting that PACSIN/EHD removal from the CPM may occur via tubular membrane formation. Interestingly, these membrane tubules were specifically enriched in the CPM region, and were not prominently observed in other areas of the cell (Supplementary Figure 4a, e, f). To investigate membrane tubulation at the CPM, we performed three-dimensional focused ion beam-scanning electron microscopy (FIB-SEM) combined with correlative light and electron microscopy (CLEM)^35^ (Fig. 4a). CLEM enabled the detection of ciliary structures with associated membrane tubules (Fig. 4b–g, Supplementary Movie 2, Supplementary Table 2). Strikingly, GFP-EHD1-positive membrane tubules were shown to be connected to the CPM. Together, our CLEM/FIB-SEM and live imaging studies confirm that membrane tubules develop from the CPM.

We examined if these PACSIN- and EHD-positive tubules contain ciliary membrane-associated cargo. ARL13B (Fig. 3f, Supplementary Figure 4g) and SMO (Fig. 3b, c, g) were not detected in PACSIN2 and/or EHD1 positive CPM tubules, whereas RAB8A was always present (Fig. 3e). Live cell imaging confirmed that fluorescent protein fusions of RAB8A are associated with CPM tubules, and co-localize with GFP-EHD1 during its removal from the CPM (Fig. 3h, Supplementary Figure 4f, Supplementary Movie 1). However, CPM localized GFP-LPAR1 was absent from membrane tubules, suggesting protein access is restricted to these structures (Supplementary Figure 4i). Overall, our findings demonstrate that PACSIN/EHD-positive CPM tubules are associated with the trafficking of specific ciliary proteins, such as the ciliogenic factor RAB8.

### PACSINs function at the CV stage before CP110 loss

**Fig. 5:**
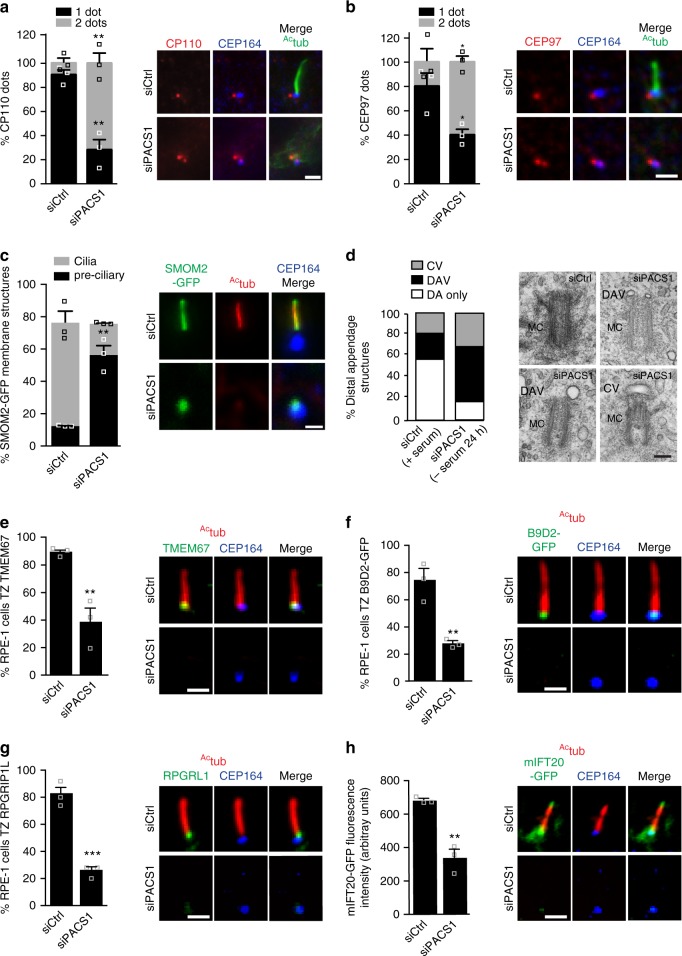
PACSIN1 functions at the CV stage prior to CP110 loss. **a** Quantification of CP110 dots in serum starved RPE-1 cells treated with siRNAs for 72 h, serum starved for the last 24 h, and stained with CP110, ^Ac^tub and CEP164 antibodies. CP110 localization on both centrioles (2 dots) or only the daughter centriole (1 dot) was quantified (left panel, siCtrl = 100, siPACS1 = 63 cells). **b** Quantification of CEP97 dots in cells treated as in **a** and stained with CEP97, ^Ac^tub, and CEP164 antibodies. CEP97 localization on the centrioles was quantified as in **a**(siCtrl = 201, siPACS1 = 125 cells). **c** Quantification of SMOM2-GFP centrosomal accumulation in cells treated as in **a**, and stained with ^Ac^tub and CEP164 antibodies. “Preciliary” indicates SMOM2-GFP vesicle accumulation at the MC without axonemes (siCtrl = 100, siPACS1 = 67 cells). Scale bar: 2 μm for images in **a**–**c**. **d** Quantification of MC with non-ciliary distal appendages structures from cells treated as in **a**. Pooled data from 3 independent experiments (siCtrl = 56, siPACS1 = 41 cells). Representative electron micrographs of quantified MCs shown on right. Scale bar: 200 nm. MC mother centriole, DAV distal appendages vesicles, CV ciliary vesicle, or DA non-membrane-associated distal appendages. **e**–**g** Quantification of TZ accumulation of TMEM67 (**e**, siCtrl = 113, siPACS1 = 89 cells), B9D2-GFP (**f**, siCtrl = 365, siPACS1 = 250 cells), RPGRIP1L (**g**, siCtrl = 70, siPACS1 = 63 cells) in cells treated as in **a**. **h** mIFT20-GFP cells treated as in **a**, stained with ^Ac^tub and CEP164 antibodies, and quantified for mIFT20 fluorescence intensity at the MC/BB (siCtrl = 46, siPACS1 = 43 cells). Scale bar: 2 μm for images in **e**–**h**. In **a**–**c**, and **e**–**h**, representative images of quantified cells shown on right and means ± SEM, *n* = 3 independent experiments. Two-tailed *t*-test analysis as compared with siCtrl was performed. **P < 0.001, ***P < 0.0001

To understand PACSIN1 function in ciliogenesis, we investigated its role in the removal of the CP110/CEP97 complex, which occurs prior to CV assembly, and requires EHD1. We found that 72 ± 6% (±SEM) of serum starved PACSIN1 depleted cells had CP110 localized to both centrioles, whereas control cells had only 9 ± 3% (±SEM) (Fig. 5a). Similarly, CEP97 was not removed in 60 ± 5% (±SEM) of PACSIN1 depleted cells compared to 20 ± 11% (±SEM) in control cells (Fig. 5b). These results indicate that PACSIN1 is required for uncapping the MC prior to axonemal growth. We next examined, in PACSIN1 depleted cells, the trafficking and accumulation of preciliary vesicles labeled with a mutant SMO (SMOM2-GFP) at the MC^8^. SMOM2-GFP membrane accumulation at the MC was not affected by PACSIN1 depletion (Fig. 5c). This result was confirmed by transmission electron microscopy (TEM), where the majority of PACSIN1 ablated non-ciliated cells had DAVs (53%) and CVs (31%) associated with the MC, structures observed to a lesser extent in serum-fed RPE-1 cells (DAVs, 26%; CVs, 17%) (Fig. 5d). Finally, PACSIN1 depletion reduced TZ proteins TMEM67, B9D2, RPGRIPL1, and the intraflagellar protein (IFT) mIFT20-GFP recruitment to the MC (Fig. 5e–h). Taken together, these results indicate that PACSIN1, similar to EHD1^8^, regulates the uncapping of the MC distal end, prior to TZ assembly and IFT recruitment upstream of axonemal growth.

### Membrane tubules connect MC and PM during ciliogenesis

**Fig. 6:**
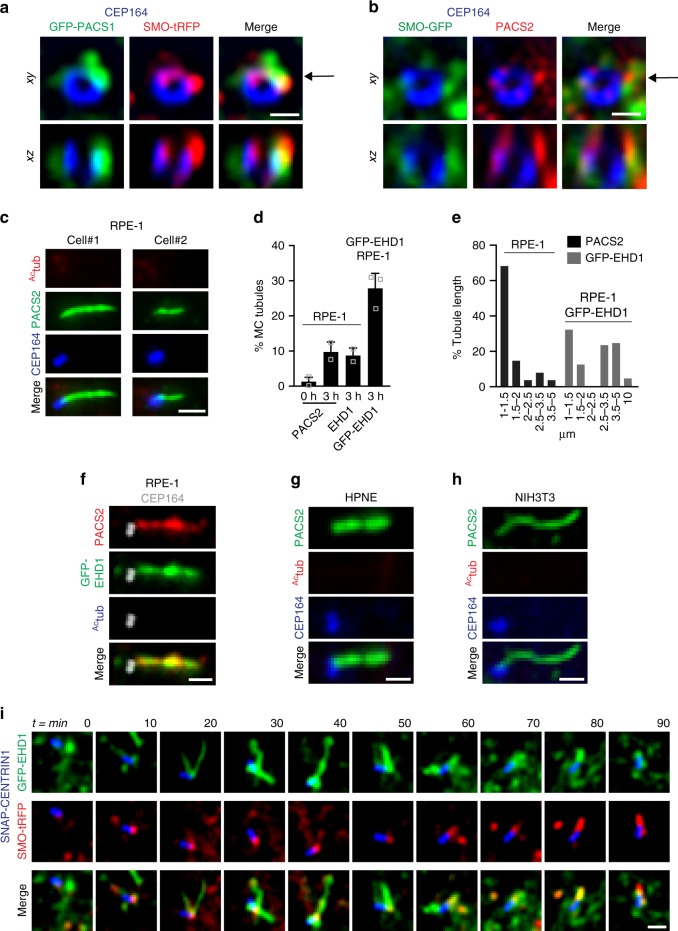
PACSIN and EHD proteins co-localize on dynamic MC-tubules during ciliogenesis. **a** Representative N-SIM images of SMO-tRFP cells transiently expressing GFP-PACSIN1, serum starved for 3 h, and stained with CEP164 antibody. **b** Representative N-SIM images of SMO-GFP cells serum starved for 3 h and stained with CEP164 and PACSIN2 antibodies. The xz images (bottom panels) in **a** and **b** show orthogonal views at the position of the arrow indicated in the *xy* plane (top panels). Scale bars: 500 nm. **c** Representative images of RPE-1 cells serum starved for 3 h and stained with CEP164, ^Ac^tub and PACSIN2 antibodies. Images were taken by epifluorescence microscopy using a 63× objective. Maximum intensity projections of deconvolved z-stacks are shown. **d** Quantification of PACSIN2, EHD1, or GFP-EHD1-positive MC tubules in RPE-1 cells, serum starved at 0 and 3 h and stained with PACSIN2, EHD1 antibodies, or observed in GFP-EHD1 cells imaged as in **c** (PACS2 0 h = 79, PACS2 3 h = 140, EHD1 = 67 cells, pooled from *n* = 2; GFP-EHD1 = 100 cells, pooled from *n* = 3). Means ± SD. **e** Graph representing the length of PACSIN2 and GFP-EHD1-positive tubules in cells treated as in (**c**) (25 tubules per condition). **f** GFP-EHD1 cells serum starved for 3 h, stained with PACSIN2, ^Ac^tub (Alexa 305 nm), and CEP164 (Alexa 647) antibodies, and imaged by epifluorescence microscopy using a 63× objective. Z-stack images were deconvolved and a single *xy* plane is shown. Note the co-localization of PACSIN2 and GFP-EHD1 in MC-associated tubules (25 cells). **g**, **h** HPNE (**g**) and NIH3T3 (**h**) cells serum starved for 3–6 h and stained with antibodies for PACSIN2, CEP164, and ^Ac^tub. Images were taken with a 100× objective and are maximum intensity projections of deconvolved z-stacks. **i** Triple line starved for 3 h, labeled with 300 nM SNAP-Cell647-SiR substrate for the last hour, washed, and imaged live every 10 min. Images are single *xy* planes (15 cells). Scale bars: 1 μm for (**c**, **f**–**i**)

Because PACSIN1 functions at the CV stage, we investigated whether these proteins are recruited to the MC at early ciliogenesis stages. GFP-PACSIN1 and PACSIN2 were detected on membrane structures near the MC distal end marked by CEP164 and partially co-localized with SMO-positive vesicles prior to ciliary growth (Fig. 6a, b). Interestingly, PACSIN2 was also observed on 1–5 μm tubular structures near the MC in ~10% of unciliated cells following ciliogenesis initiation (3 h serum starvation) (Fig. 6c–f, Supplementary Figure 5a). This tubular localization was also observed in 11% HPNE (238 cells) and 2.5% NIH3T3 (46 cells) of unciliated cells following 3–6 h serum starvation to induce ciliation (Fig. 6g, h, Supplementary Figure 5b, c). As in CPM tubules, GFP-EHD1 and PACSIN2 co-localized on all MC-associated membrane tubules (Fig. 6f), and these structures were specifically enriched at the MC and typically were not observed elsewhere in the cell (Supplementary Figure 5a–e). Overexpression of GFP-EHD1 enhanced the frequency and length of these tubules specifically at the MC (Fig. 6d, e, Supplementary Figure 3f, and 5d, e). By time-lapse imaging and using a cell line expressing GFP-EHD1, SNAP-CENTRIN1 to mark the centrioles, and SMO-tRFP (triple line), we determined that membrane tubules assemble and disassemble during ciliary formation (Fig. 6i). These findings indicate that PACSINs localize to preciliary membranes, likely DAVs and/or CVs, and are associated with dynamic membrane tubules near the MC along with EHD1 during ciliogenesis.

**Fig. 7:**
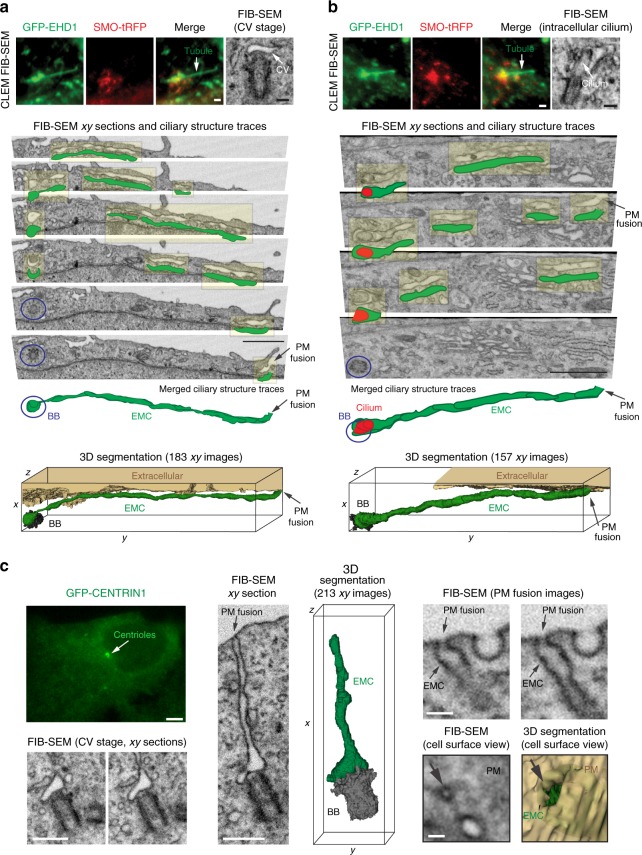
Membrane tubules connect the CV/developing cilium and the PM creating an extracellular membrane channel. **a**, **b** CLEM/FIB-SEM analysis of a 3 h starved GFP-EHD1 + SMO-tRFP cell showing a CV (**a**) and a short intracellular cilium (**b**) connected to GFP-EHD1-positive tubular membranes. Fluorescence images (upper left and middle panels, scale bar: 1 μm) and longitudinal FIB-SEM section of the BB/cilium (upper right panel, scale bar: 200 nm). Below are representative FIB-SEM xy image sections (183 sections in **a** and 157 sections in **b**) showing a continuous membrane tubule connecting the CV and short intracellular cilium to the PM, respectively (Scale bar: 1 μm). Blue circles highlight the BB region. Traces for the extracellular membrane channel (EMC) connected to the CV (**a**) or short intracellular cilium (**b**) are shown in green offset from FIB-SEM EMC structures (highlighted in yellow). The cilium outline is traced in red. Merged ciliary structure traces and 3D segmentation of the CV and short intracellular cilium and the EMC connected to the PM from the xy FIB-SEM images are shown in the last two lower panels. The raw FIB-SEM images for **a** can be found in Supplemental Movie 3 and **b** in Supplemental Movie 4. **c** CLEM/FIB-SEM of 3 h starved GFP-CENTRIN1 cell (fluorescence images in upper left panel, scale bar: 2 μm). Representative FIB-SEM images (213 xy images) (bottom left and middle left panels, scale bars: 500 nm) and 3D segmentation (middle right panel) showing an EMC connecting the CV and the PM. FIB-SEM images (right panels, scale bar: 100 nm) showing different cross-sections of the EMC connected to the plasma membrane (PM) (top right panels). A view of the cell surface showing the EMC opening at the PM (arrow) and the 3D segmentation of the EMC opening (green) at the PM (bottom right panels). A summary of CLEM FIB-SEM data sets is shown in Supplementary Table 2

To further investigate the relationship between MC-associated membrane tubules and ciliary assembly, we performed CLEM/FIB-SEM with cells expressing GFP-EHD1 and SMO-tRFP following the promotion of ciliogenesis. Remarkably, GFP-EHD1-positive membrane tubules were attached to either CV membranes (3 of 7 cells), short intracellular cilia sheath membranes (2 of 7 cells), or mature cilia CPM (2 of 7 cells) (Fig. 7a, b, Supplementary Table 2, Supplementary Movie 3 and 4). These results demonstrate that PACSIN and EHD proteins specifically mark membrane tubules attached to early intracellular ciliary membrane structures. Surprisingly, 3 out of 5 FIB-SEM imaged cells undergoing intracellular ciliogenesis demonstrated CVs or ciliary sheaths with tubule membrane connections to the PM, creating a continuous open channel to the extracellular space, hereafter referred to as an EMC (Fig. 7a, b, Supplementary Table 2). In other cells with tubules, the PM connections were not observed or difficult to resolve by FIB-SEM, suggesting that EMCs have not yet been established. To rule out that the formation of MC-associated membrane tubules and EMCs was due to higher expression of GFP-EHD1 compared to the endogenous protein (Supplementary Figure 3f), we performed CLEM/FIB-SEM on 3 h serum starved RPE-1 cells expressing near endogenous levels of GFP-EHD1 (Supplementary Figure 6a) or GFP-CENTRIN1 cells and discovered 3 out of 3 cells and 3 out of 19 cells from these lines, respectively had tubules >1 um attached to CVs and/or short cilia (Fig. 7c, Supplementary Figure 6b, Supplementary Table 2). These MC attached structures could also be observed using CLEM with serial section TEM (Supplementary Figure 6c, Supplementary Table 2). Similar to what was observed with higher ectopic expression of GFP-EHD1, EMCs were observed in 2 out of 3 cells expressing near endogenous levels of GFP-EHD1 and remarkably, a ~1.5 μm EMC was clearly identified in one of the GFP-CENTRIN1 cells imaged by FIB-SEM (Fig. 7c, Supplementary Movie 5). The EMC connection between the developing intracellular cilium and PM was further shown by treating cells with the transition metal ruthenium red (RR), which enhances the EM contrast of extracellularly exposed membranes (Supplementary Figure 6b–d). Together, these unexpected findings suggest that membrane tubulation is important for fusing the developing intracellular ciliary membrane with the PM.

### MC-membrane tubule formation requires PACSIN and EHD proteins

**Fig. 8:**
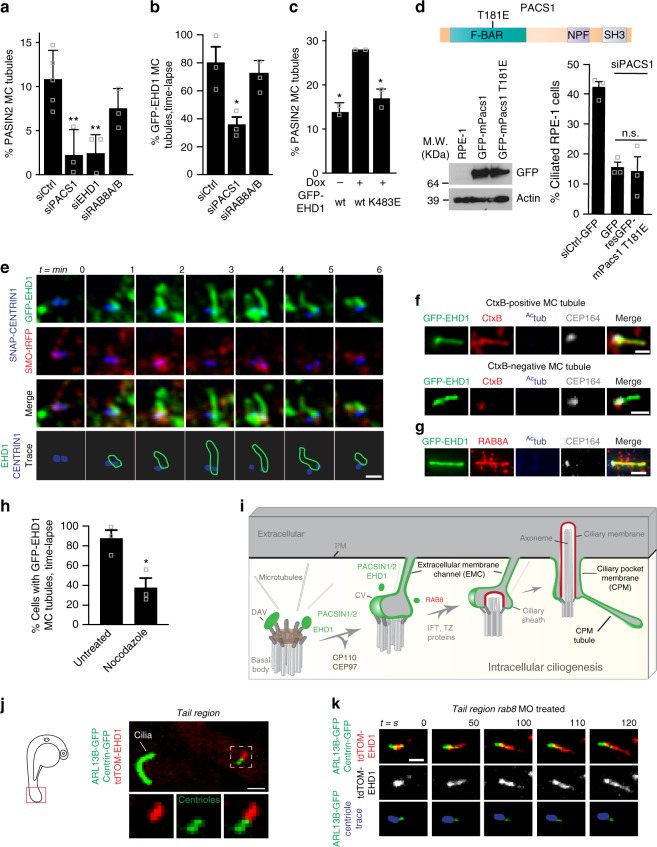
MC-membrane tubules are PACSIN1-, EHD1- and MT-dependent and form in vivo. **a** Quantification of PACSIN2 positive MC-tubules in cells as in Fig. 6c following siRNAs treatments (siCtrl = 258 cells, *n* = 5; siPACS1 = 168, siEHD1 = 75, siRAB8A/B = 147 cells, *n* = 3). **b** Quantification of GFP-EHD1-positive MC-tubules from triple line treated with siRNAs, starved 3 h and imaged every 2 min for 30 min (siCtrl = 35, siPACS1 = 36, siRAB8A/B = 57 cells, *n* = 3). **c** Quantification of MC-tubules from cell lines starved for 3 h and stained with PACSIN2 and CEP164 antibodies (wt −dox = 262, wt +dox = 142, K483E +dox = 177 cells, *n* = 2). **d** Schematic of the T181E tubulation defective mutation in the F-BAR domain of mPacsin1 (top). Immunoblot analysis of cells transfected with wildtype- or T181E-mPacsin1 after 6 h of siRNA treatment (bottom left) and ciliation rescue experiment (bottom right, siCtrl = 108, GFP = 71, rescT181E = 85 cells, *n* = 3). **e** Images of the 3 h starved triple line taken every minute. Membrane tubules are outlined in green and centrioles in blue (bottom panel, 15 MC-tubules). Single *xy* planes were smoothed. **f** Single plane xy epifluorescence images of 3 h starved GFP-EHD1 cells that were CtxB positive (~5% of cells). 53% of GFP-EHD1 MC-tubules contained CtxB (12 MC-tubules). **g** Images of GFP-EHD1 cells as in **f** stained with RAB8A, ^Ac^tub, and CEP164 antibodies (10 cells). Scale bars: 1 μm in (**e**-**g**). **h** Quantification of cells as in **b** treated with 10 μM Nocodazole (untreated = 38, Nocodazole = 40 cells, *n* = 3). **i** Model for intracellular ciliogenesis. DAV distal appendage vesicle, CV ciliary vesicle, IFT intraflagellar transport, TZ transition zone, PM plasma membrane. In **a**–**d** and **h**, means ± SEM and two-tailed *t*-test analyses are indicated in figure. *P < 0.05, **P < 0.001, non significant (n.s). **j** Live image of the tail (red box, left schematic) from a 24 hpf embryo showing a tdTom-EHD1 positive MC-tubule (white box, 9 MC-tubules). **k** Timelapse of MC-tubule formation (centrioles in blue) as in **j** from embryo injected with *rab8* MO (11 MC-tubules). Scale bars: 2 μm in **j** and **k**

Next, we investigated requirements for ciliogenic membrane tubulation in fixed RPE-1 cells and the live RPE-1 “triple line” following RNAi treatment. PACSIN2-positive tubules were strongly diminished at the MC following PACSIN1 and EHD1 ablation (Fig. 8a). Likewise, PACSIN1 depletion reduced GFP-EHD1 tubule detection and frequency by more than half (36 ± 5% (±SEM)) compared to siCtrl-treated cells (80 ± 11% (±SEM)) during the imaging time course (Fig. 8b, Supplementary Figure 7a). These results suggest that PACSIN1 and EHD1 are essential for membrane tubulation during ciliogenesis. A functional requirement for EHD1 in the formation of MC-associated membrane tubules during early stages of ciliogenesis is supported by the observation that these structures were increased by the expression of wildtype GFP-EHD1 but not by a tubulation defective GFP-EHD1 K483E mutant^36^ (Figs. 6d and 8c, Supplementary Figure 3f). Previously, we demonstrated that this mutant is expressed at similar levels as GFP-EHD1 and localizes to DAVs, yet does not rescue ciliogenesis in EHD1 ablated cells^8^. Together, these results support a role for EHD1, post-CV-stage, in promoting tubulation of the developing intracellular ciliary membranes for the establishment of EMCs. To test if the tubulation properties of the PACSIN1 F-BAR domain are required for ciliation, we performed RNAi rescue experiments with murine T181E Pacsin1, a mutant unable to tubulate membranes^37^. Unlike WT Pacsin1 (Fig. 1c), the T181E mutant failed to promote ciliation following PACSIN1 siRNA treatment (Fig. 8d) indicating that membrane tubulation properties of PACSIN1 are critical for ciliation.

Because PACSINs localize to early ciliary membrane structures, but are also found on the PM^23^, we next asked whether membrane tubules originate from the MC or PM. Live imaging of the “triple line” at 1 min intervals revealed that GFP-EHD1 tubules originate and grow away from the MC (Fig. 8e, Supplementary Movie 6), suggesting that the developing intracellular cilium initiates membrane tubule connections with the PM. Consistent with this observation Cholera Toxin B (CtxB), which has been shown to stain the PM and PACSIN2-positive caveolar membrane tubules in unpermeabilized cells^38^, co-localized with approximately half of GFP-EHD1 tubules associated with the MC (Fig. 8f, Supplementary Figure 7b). Likewise, not all MC-associated membrane tubules displayed stronger RR staining compared to cytosolic membranes (Supplementary Figure 6c). Together, these studies indicate that ciliogenic membrane tubulation occurs from the developing intracellular ciliary membrane to establish EMCs.

Next, we investigated whether ciliogenic membrane tubules are associated with proteins targeted to the growing cilium. Two proteins known to accumulate in developing ciliary membranes, SMO-GFP and ARL13B^8,39,40^ were not observed in these tubules (Figs. 6i, 7a, b, and 8e, Supplementary Figure 7c, d). In contrast, endogenous and ectopically expressed RAB8A were detected and always co-localized with GFP-EHD1-positive tubules near the MC (Fig. 8g, Supplementary Figure 7c, d). However, unlike PACSIN1 or EHD1 depletion, RAB8A/B knock-down did not affect the formation of MC-associated membrane tubules (Fig. 8a, b). Together, our results support a model wherein PACSIN1 and EHD1 function in membrane tubulation during early stages of ciliogenesis necessary for EMC formation, a process that appears to be associated with RAB8 recruitment to the growing cilium.

Because MC-associated membrane tubules and EMCs observed by EM followed a relatively linear path toward the PM, we theorized that microtubules guide them. As predicted, treatment of cells undergoing ciliogenesis with the microtubule inhibitor nocodazole reduced the frequency of these structures (Fig. 8h, Supplementary Figure 7e). Together, our findings indicate that membrane tubules originate from the CV/ciliary sheath membrane and require microtubules to grow towards the cell surface, where these membrane structures fuse to form the EMC (Fig. 8i).

### MC-membrane tubules form during ciliogenesis in vivo

Finally, we investigated whether membrane tubulation is associated with ciliogenesis in vivo in zebrafish. We examined the tail region of 24 hpf embryos since *pacsins* are required for ciliogenesis in these cells (Supplementary Figure 2d) and EHD1-positive CPM tubules were detected (Fig. 3f). Using the *Tg*(*centrin:GFP*) transgenic line injected with mRNA for the ciliary marker ARL13B-GFP and tdTom-EHD1, it was possible to monitor both ciliated and unciliated cells by live SDC imaging (Fig. 8j). Remarkably, tdTom-EHD1 membrane tubules were detected on the MC of unciliated cells. Due to high level of ciliation in these cells, we co-injected zebrafish embryos with a *rab8* morpholino (MO) to block ciliary axoneme growth^41^ and facilitate our analysis of MC-membrane tubules at early ciliogenesis stages. Ciliation was dramatically reduced by *rab8* MO treatment (Supplementary Figure 7f), and tdTom-EHD1 membrane tubules were also detected developing from the MC (Fig. 8k). Together, these results demonstrate that MC-associated membrane tubules are observed during early ciliogenesis in vivo, further supporting a model where PACSINs and EHDs function to assemble these structures during ciliogenesis.

## Discussion

Here, we demonstrate that the F-BAR proteins PACSIN1 and -2 are not only critical for CV assembly but along with EHD1 also function in the formation of membrane tubules that connect the intracellular developing cilium to the cell surface. Our findings explain how intracellular cilia are able to fuse with the PM and emerge from the cell to contact the extracellular signaling space. We also discovered the existence of PACSIN- and EHD-associated membrane tubules that develop from the CPM and contain the ciliogenic factor RAB8. Finally, our work shows that these membrane tubulating proteins have similar properties in cultured human cells and in vivo in zebrafish embryos.

EHD1 is known to recruit PACSINs to cellular membranes^23^. Consistently, whenever ciliary-associated EHD1 was observed, PACSIN1 or -2 was co-localized, with overexpression of GFP-EHD1 promoting membrane tubulation on the developing intracellular cilium, but not the tubulation defective K483E mutant. Importantly, EHD and PACSIN proteins showed specific enrichment on ciliary-associated membrane tubules. In endosomes, PACSIN2 remodels EHD1-positive vesicles and causes reduced membrane tubulation upon depletion^42^. Our data support a similar function for PACSIN1 and -2 at the developing cilia and the CPM. During ciliogenesis, PACSIN and EHD proteins are recruited to DAVs, where they function to assemble the CV. This ciliogenesis step is required to remove the CP110/CEP97 cap from the MC and for the recruitment of ciliary TZ and IFT proteins. Our findings suggest that CV assembly involves PACSIN-mediated membrane reshaping to promote fusion of DAVs, in conjunction with EHD proteins and SNAREs^8^. Amid CV assembly, PACSINs and EHDs form dynamic membrane tubules, which are guided by microtubules to the PM where these membranes fuse to establish the EMC. In vivo studies of *Pacsin1* or *-2* knockout mice do not report ciliopathy defects and mice are viable^43^. Our findings suggest that *PACSIN1* and *-2* have ciliogenic, albeit tissue-specific, functions. Therefore, single knockout approaches in mice could result in milder phenotypes due to compensatory functions of the two genes. In zebrafish, CRISPR/Cas9 *pacsin* mutant embryos revealed that despite inherent mosaicism (Supplementary Figure 2e, f), the strong tissue-specific ciliary phenotypes were faithful consequences of our genome editing-approach.

Interestingly, PACSIN and EHD^8^ proteins do not accumulate in the ciliary membrane, but it stands to reason that ciliary exclusion of the positive-membrane curvature-sensing PACSIN proteins after the CV stage, in the growing cilium, may be necessary to establish and maintain a negative ciliary membrane curvature inside the developing organelle. This is consistent with the timing of TZ assembly following CV formation upstream of RAB8-dependent axonemal growth^8^. PACSINs have unique membrane sculpting properties that differentiate them from other F-BAR domain proteins, and allow them to produce a large spectrum of membrane tubules with varying diameters^20^. In particular, PACSINs possess the ability to bind membranes of shallower curvature in vitro and in vivo, as shown for Pacsin1 during activity-dependent bulk endocytosis (ADBE)^20,44^. These membrane binding properties could allow PACSIN1 and -2 to function with various membrane shapes ranging from smaller DAVs, the larger CV, the ciliary sheath, and CPM (Fig. 8i). PACSIN assembly into tip-to-tip oligomeric scaffolds is promoted by an increase in local protein concentration and specific membrane curvatures^45,46^. Therefore, both these factors could facilitate the F-BAR domain oligomeric assembly during ciliogenesis and in the CPM. Furthermore, trafficking and accumulation of PACSINs in ciliary-associated membranes is likely regulated by lipids. Indeed, PACSINs generate tubules from PI(4,5)P2-enriched membranes^21^, which localize to the CPM base^47–50^. Interestingly, PI(4,5)P2 is absent from the cilium, and therefore could be associated with PACSIN restriction from this organelle.

The discovery that membrane tubulation is important for the fusion of the developing cilium with the cell surface offers new insight into the intracellular ciliogenesis pathway. We provide evidence of a rarely described cellular phenomenon whereby intracellular membrane tubules fuse with the PM. A similar process occurs from late endosomes and lysosomes in invadopodia and during HIV release, respectively^51,52^. In the latter example, 3D-SEM technology was used to identify “virion channels”. Here, we have shown direct ciliary association and characterization of EMCs and CPM tubules using FIB-SEM. Indeed, characterization of the EMC was not possible with traditional methods such as serial sectioning TEM (Supplementary Figure 6c). Our work suggests that during ciliogenesis, the establishment of membrane tubules is involved in directing proteins such as RAB8, and possibly lipids, to the developing cilium. PACSIN accumulation within tubular membranes could provide a molecular identity to the EMC and regulate membrane carrier transport, a function described for some BAR proteins^45^. PACSINs also regulate actin and microtubule cytoskeleton assembly^24,53^. Thus, constant interplay of PACSINs with the cytoskeleton may provide a force-generating role for the establishment of EMCs and guidance for fusion with the PM.

This work also sheds new light on the CPM structure and function. In the PM, membrane tubules are important for clathrin-independent endocytic trafficking^54^. Thus, the presence of PACSIN- and EHD-positive CPM tubules may not be unexpected given the known function of these proteins in endocytic pathways at the PM^25,26,38,55^ and the continuity between the CPM and the PM, both sites for clathrin- and caveolin-mediated endocytosis^6,56^. Our findings show that PACSIN and EHD proteins have dynamic CPM localization, suggesting an important role in protein trafficking to and from the CPM. The localization of these proteins at the CPM could contribute to lateral membrane diffusion restrictions important for regulating ciliary transport^29,57^, an attractive concept worth investigating. Interestingly, besides RAB8, other ciliary membrane-associated proteins observed were absent from CPM tubules, suggesting that these structures have selective cargo sorting. Indeed, the specification of cargo is supported by our observation that PM- and CPM-localized GFP-LPAR1 is not detected in these membrane tubules. Interestingly, RAB8 levels are reduced in the cilium following ciliogenesis, presumably as a length control mechanism^13^, suggesting that CPM tubules may be important for this process. Our work also supports observations from a recent report by Saito et al.^58^, in which membrane tubules were detected near the disassembling cilium. CPM tubules would have a high capacity to remove large quantities of cargo rapidly during this process. Together, our findings reveal a critical role for membrane shaping proteins in tubulation processes important for intracellular ciliogenesis and ciliary trafficking. Exploring these processes further is expected to enhance our understanding of ciliogenesis and ciliary signaling in normal and diseased contexts.

## Methods

### Antibodies and reagents

Commercial antibodies used were as follows: anti-Acetylated tubulin (^Ac^tub, clone 6-11B-1, 1/10000, T6793, Sigma), anti-Gamma-tubulin (GTU-88, 1/1000, T6557, Sigma), anti-b-actin (clone AC-15, 1/30000, A5441, Sigma), anti-PACSIN1 (1/100, 196 003, Synaptic Systems), anti-PACSIN2 (1/250, ab37615, Abcam), anti-PACSIN2 (1/500, 10518-2-AP, Proteintech), anti-PACSIN3 (1/100, ab37612, Abcam), anti-Pericentrin (PCTN, 1/5000, NB100-61071, Novus Biologicals), anti-EHD1 (EPR4954, 1/500, ab109311, Novus Biologicals), anti-RPGRIP1L (1/200, 55160-1-AP, Proteintech), anti-TMEM67 (1/200, 12780-1-AP, Proteintech), anti-CEP164 (1/500, sc-240226, Santa Cruz), anti-CP110 (1/1000, 12780-1-AP, Proteintech), anti-CEP97 (1/1000, A301-945A, Bethyl), anti-Arl13b (1/300, clone N295B/66, NeuroMab/UC Davis), anti-GFP Alexa 488 (1/1000, A21311, Molecular Probes Life Technologies), Phalloidin conjugated with Alexa 488 (1/50, A12379, Molecular Probes Life Technologies), Hoechst (1/3000, H3570, Molecular Probes Life Technologies) and all secondary antibodies were from Life Technologies. The rabbit anti-RAB8A antibody was a gift from Johan Peränen (University Helsinki, Finland). SNAP-Cell647-SiR reagent was purchased from New England Biolabs. Nocodazole (Calbiochem) was from Sigma. Doxycyclin hydrochloride was obtained from Sigma and used according to manufacturer’s instruction.

### Cell lines, plasmids, and RNAi

Human (hTERT-RPE, PANC1, hTERT-HPNE, NIH3T3, and NeoHFF) and mouse IMCD3 cell lines were obtained from ATCC. *hPACSIN1* (BC040228), *hPACSIN2* (BC008037), *hPACSIN 3* (BC007914), *LPAR1* (NM_001401), *CENTRIN1* (BC029515), *ARL13B* (BC094725) complementary DNAs were purchased from DNASU. Murine *Pacsin1* was obtained from Transomic Technologies (BC014698). tRFP-RAB8A was previously described^13^. The *mIFT20* construct was as previously mentioned^8^ and subcloned into pGLAP7 to generate a stable mIFT20-GFP RPE-1 line using the Flp-In system (Invitrogen) as described^8^. *T181E-Pacsin1* mutants were generated using the QuikChange mutagenesis kit (Agilent Technologies) as described in Quan et al.^37^. cDNAs were cloned into Gateway compatible entry vectors and sub-cloned into pGLAP1, pCS2+, or the inducible lentivirus expression vector pHUSH-LAP as previously published^8^. SMO-GFP, SMOM2-GFP, SMO-tRFP, GFP-B9D2, GFP-EHD1, GFP-EHD1K483E, and GFP-RAB8A RPE-1 cell lines were as previously described^8,59^. *GFP* and *SNAP* tagged *CENTRIN1* were subcloned into the pHUSH-Ubc lentivirus expression system. GFP-EHD1, GFP-PACSIN -1, -2 and -3, GFP-EHD1 + SMO-tRFP, and the triple (GFP-EHD1 + SMO-tRFP + SNAP-CENTRIN1) RPE-1 cell lines were generated using lentiviral infection as previously published^8^. X-treme Gene 9 (Roche) was used for DNA transfections into cells. Gene expression in lines created with the inducible lentivirus system was controlled using 1 μg per ml of doxycycline unless specified. For knockdown experiments, cells were transfected with siRNA duplexes obtained from Dharmacon (Supplementary Table 1) using RNAiMAX (Invitrogen) according to manufacturer’s instruction and harvested after 72 h treatment unless indicated otherwise in figure legend. siRAB8A and siRAB8B have been described^13^. For zebrafish experiments, *tdTom-EHD1* and *ARL13B-GFP* cDNAs were subcloned into pCS2+ vectors.

### Immunofluorescence and time-lapse microscopy

All human (hTERT-RPE, PANC1, hTERT-HPNE, NIH3T3, and NeoHFF) and mouse IMCD3 cell lines were serum starved for 24 h for ciliation assays and 3–6 h for early ciliogenesis analyses, followed by fixation, and immunostained with ^Ac^tub and PCNT antibodies and Hoechst as described^8^ or as indicated in figure legends. Briefly, fixation was performed using 4% paraformaldehyde or cold methanol for 10 min, followed by blocking for 10 min with 1% BSA in PBS 0.1% Triton X-100, and immunostaining in blocking solution. More than six fields per condition were imaged using a 40× 1.4 numerical aperture (NA) or 63× 1.3 NA objective (Zeiss objectives) and a Zeiss Axio Scan Z1 inverted epifluorescence microscope equipped with an X-Cite (120 Series) lamp and a CoolSNAP HQ2 camera (Photometrics). All confocal images were taken using a Marianas spinning disc confocal microscope (Intelligent Imaging Innovations) equipped with 40× 1.4 NA, 63× 1.3 NA, or 100× TIRF 1.46 NA Zeiss objectives as indicated in figure legends and EMCCD Evolve 512 (Photometrics). Alternatively, a CMOS (Hamamatsu) camera was used as indicated in figure legend. Nearest-neighbor deconvolution was applied with SlideBook software when indicated in the figure legend. Images and fluorescence intensities were analyzed with the SlideBook software. Measurements of mIFT20-GFP fluorescence intensity were done as previously described^8^. SNAP-Cell647-SiR (New England Biolabs) substrate was used for cellular labeling according to manufacturer’s recommendation and as described in figure legends. For Cholera Toxin B experiments, Alexa-Fluor-555-labeled CtxB B (Invitrogen) was diluted at 2 μg per ml in starvation medium. GFP-EHD1 RPE-1 cells were incubated for 5 min at 37° in the presence of the toxin prior to fixation with 4% PFA/PBS as described above and staining with ^Ac^tub, and CEP164 antibodies.

For whole-mount zebrafish cilia studies, embryos were fixed at 72 hpf after a brief pre-permeation incubation for 90 s into a solution of 0.5% Triton X-100 or directly incubated in 4% PFA/PBS for 4 h at room temperature followed by immunostaining with ^Ac^tub antibody, phalloidin, and Hoechst as described previously^8^ unless specified otherwise in figure legend. Longer incubation times of 18–24 h were used with the anti-Pacsins primary antibodies to promote penetration. Images (35 μm z-stacks with 1 μm step size) were acquired using the Marianas SDC microscope and 40 × 1.4 NA objective. For zebrafish cilia quantification, number of ciliated organs with absent or strongly reduced cilia were used to calculate the percentage of abnormal organs as previously described in Lu et al.^8^.

Time-lapse imaging of fluorescently-tagged proteins was performed as previously described^8^ and as specified in figure legends. For zebrafish time-lapse imaging, 24 hpf embryos were anesthetized in a solution of tricaine (MS-222, Sigma Cat# A-5040, 150 mg per L), placed in a Lab-Tek chamber (Cat# 4802). Embryos that expressed optimal amounts of fluorescence for all proteins of interest were screened and imaged at room temperature using the Marianas SDC microscope and 63 × 1.3 NA objective at room temperature. All images are maximum intensity projections from a z-stack.

TIRF imaging used a Zeiss Axio scan Z1 microscope equipped with the CoolSNAP HQ2 camera (Photometrics) and a 100 × 1.46 NA oil TIRF objective (Zeiss) and controlled by SlideBook software. Cells were placed into a temperature-controlled chamber set at 37° and 5% CO_2_. TIRF excitation was performed using a 488 and 561 nm solid-state lasers and a Zeiss TIRF slider. The TIRF angle for each channel was adjusted in live mode until most of the cytoplasmic background signal disappeared and only the target ciliary structure was visible. Images were taken in a single focal plane every 15 s for 15 min with Definite Focus (Zeiss).

Smooth function was applied using ImageJ to some images as indicated in figure legend.

### Structural illumination microscopy (SIM)

Coverglasses (no. 1.5) with fixed cells incubated with 100 nm Tetra Speck beads (Life Technologies) in the final wash were mounted onto glass slides with Vectashield H-1000 (Vector) and sealed with nail polish. All 3D-SIM imaging was performed using a Nikon N-SIM (Nikon Instruments, Melville, NY) or a Zeiss SIM (Carl Zeiss Microscopy, LLC, Peabody, MA) microscope as specified in figure legend. Nikon SIM images were taken with a SR Apo TIRF × 100/1.49 NA oil immersion objective (Nikon) and EMCCD camera (Andor DU-897E) and Zeiss SIM images were taken with a 63 × 1.4 NA oil objective (Zeiss) and a PCO edge sCMOS camera as described previously^8^. Briefly, image stacks were acquired of typically 2 μm height with 15 images per plane and a *z*-distance of 0.1 μm. Alignment parameters for all color channels were carefully determined during the calibration procedure with Tetra Speck beads. Image reconstruction and processing was performed with the Nikon Elements or Zeiss Zen software and tiffs edited in ImageJ. Intensity profiles were generated as previously described^8^.

### Stochastic optical reconstruction microscopy

Stochastic optical reconstruction microscopy (STORM) Imaging was performed as previously described^60^ on a Nikon N-STORM microscope equipped with SR HP TIRF × 100 oil lens (NA 1.49), an Andor iXon Ultra (DU-897U) camera, and a cylindrical lens for 3D reconstruction using optical astigmatism^61^. Cells expressing SMO-GFP to label the ciliary membrane were grown on 25 mm coverslips and immunostained using the anti-PACSIN2, anti-acetylated tubulin antibody, and anti-GFP directly conjugated to the CF 568 dye (Biotium) using the immunofluorescence protocol described above. We used the following secondary antibodies: anti-rabbit Alexa 647-conjugated secondary antibody and anti-mouse DyLight 350 (ThermoFisher). Coverslips were mounted in STORM MEA buffer (50 mM MEA in GLOX buffer) in a magnetic chamber (Chamlide, CM-B25-1) covered by a glass window (Edmund Optics, 02-199) sealed with plastalina modeling clay. 25,000 image frames were acquired for each color channel with 10 ms exposure time. 647 and 561 nm at 100% level were used to excite Alexa 647 and CF 568 fluorophores, respectively. Both lasers were operated at oblique illumination angles with N-STORM Zoom set on 4× to increase excitation light power. Additionally, the 405 nm laser was used to facilitate blinking of fluorophores. 100 nm fluorescent beads (TetraSpeck) where used for system calibration and chromatic correction. Images were processed and analyzed in Nikon Elements software, with sample drift corrected by cross-correlation. Wide-field images prior to STORM imaging were collected using the same settings (microscope and objective) described above with the cylindrical lens removed from the optical path, and the sample was illuminated with an Intensilight light source (Nikon).

### Transmission electron microscopy

TEM experiments were carried out as previously described^8^. Briefly, RPE-1 cells were fixed with 2.5% glutaraldehyde buffered in 0.1 M sodium cacodylate buffer overnight followed by 1% osmium tetroxide for 30 min at RT. After subsequent washes and prestaining with 0.5% uranyl acetate for 1 h at RT, cells were dehydrated in graded ethanol series and embedded in polybed 812 resin (PolyScience). Thin sections were cut using a diamond knife and imaged on an electron microscope (FEI T12) equipped with a CCD camera. Sample preparation for RR staining was performed as described previously^62^. Briefly, RPE-1 cells expressing SMO-tRFP/GFP-EHD1 treated with doxycycline (0.1 μg per ml) were fixed using 2.5% glutaraldehyde and 0.2% RR in 0.1 M cacodylate buffer, for 1 h at room temperature. After three washes in 0.1 M cacodylate buffer, samples were post-fixed in 1% osmium tetroxide containing 0.2% RR in 0.1 M cacodylate buffer for 1 h at 4 °C followed by uranyl acetate staining, dehydration in graded ethanol series, and embedding as described above. Sample blocks were used for FIBSEM or serial sectioning.

### CLEM/FIB-SEM

CLEM/FIB-SEM sample preparation: Sample preparation for CLEM and FIB-SEM (CLEM/FIB-SEM) was performed as described previously^63^. Briefly, cells were grown on alphanumerically coded gridded coverslips, and, after fixing, various cells of interest were imaged by fluorescent microscopy as described above. Immediately after fluorescence imaging, bright field images of the gridded pattern containing the cells were acquired to generate an accurate “target map” of candidate cells for interrogation by FIB-SEM. The cell samples were then post-fixed, stained, dehydrated, and embedded in PolyBed resin according to standard protocols, allowing the etched alphanumeric pattern to be transferred to the resin. The blocks were then gently cleaned, affixed to an SEM stub with conductive silver paint, and sputter coated with a thin conductive layer of carbon before transfer to the FIB-SEM instrument.

FIB-SEM data collection: FIB-SEM imaging was performed in a Zeiss Crossbeam 540 (Carl Zeiss Inc.) in conjunction with ATLAS3D software (Fibics Inc.), as previously published^35,63^, with a few modifications (manuscript in preparation). Briefly, a platinum and carbon patterned protective pad was deposited with the FIB operated at 700 pA, and data collection was executed with the FIB and SEM operated simultaneously. The FIB was operated at 30 kV, 700 pA, SEM operated at 1.5 kV, 1 nA, and back scatter signal was recorded at the in-column EsB detector operated with a 900 V grid voltage. The “ROI” images were acquired at 3 nm pixel sampling and 9 nm milling increments, with total dwell time of 3 µs per pixel. An imaging run covering portions of a cell typically lasted ~20 h and generated a stack of ~1000 high resolution images; however, the volume containing the centriolar area was much smaller. These images were registered using in-house IMOD^64^ based scripts, and subsequently cropped, binned and inverted to yield registered, isotropic (9 × 9 × 9 nm) .mrc volumes. These data provided a high-resolution 3D EM readout corresponding to the targeted cellular features imaged previously by fluorescence, and centrioles could be easily identified without further correlative fiducial markers. FIB-SEM image volumes were visualized in 3DSlicer^65^, and features of interest from these volumes were segmented and rendered using various modules in 3DSlicer.

FIB-SEM reconstruction: FIB-SEM reconstructions were analyzed using IMOD (version 4.7) and 3DSlicer (version 4.6) software. The slicer module in IMOD was used to capture 2D images in both acquisition and arbitrary planes; ImageJ, iMovie, and Wondershare Filmora were used to generate movies from these sections. Three-dimensional volume segmentation models were generated using 3DSlicer with ciliary-associated structures (centrioles, ciliary membrane, PM, and tubules) highlighted using a combination of automatic thresholding and manual assignments. Segmentation assignments were aided by checking accuracy of structures in all three planes (*xyz*) FIB-SEM image planes. 3DSlicer (screen shot tool) was used for visualization and generation of merged FIB-SEM/3D segmentation images, as indicated.

### Zebrafish embryos husbandry and injections

Fish care and husbandry were performed in strict accordance with good animal practice as defined by the relevant national and/or local animal welfare bodies, and all animal work was approved by the National Cancer Institute at Frederick Animal Care and Use Committee (Study Proposal 17-416). Zebrafish used in this study were TAB-5. For live cell imaging studies, we used the transgenic line *Tg(5actb2:cetn2-GFP)*^*cu6*^, referred to as *Tg(centrin:GFP)*, a generous gift from Brian Perkins (Cleveland Clinic, OH). *Pacsin1b* and *2* gene expressions were disrupted using the CRISPR Cas9 system. Targeting guide RNAs and PCR detection primers were designed using CHOPCHOP^66^ unless otherwise noted. gRNAs were synthesized in vitro using assembled templates^67^ as described in Supplementary Table S1. All capped mRNAs were generated from pCS2+ vectors templates using the mMESSAGe mMACHINE T3 kit (Thermo Fisher Scientific) as previously described^8^. pT3TS-nCas9n plasmid^68^ (Addgene #46757) was used as the template for Cas9 RNA and Xba I linearized. One-cell stage embryos were injected with 50–100 pg gRNA and 200–300 pg Cas9 RNA. *Pacsin* isoform gene expression was targeted as follows: *pacsin1b*-equal amounts of gRNA targeting exons 2 and 3, *pacsin2*-exon 2, and simultaneous targeting of both *pacsins*-*1b* and *2* using a single gRNA designed for a conserved *pacsin* sequence (using CRISPR MultiTargeter^69^). Rescue experiments were carried using the same amounts of gRNA and Cas9 RNA described above with 250 pg human PACSIN1 or PACSIN2 RNA. For the T7 endonuclease assay, genomic DNA was isolated from 72 h post fertilization embryos using a published method^70^. Briefly, genomic DNA was extracted by adding 25 µl 50 mM NaOH and heated at 95 °C for 10 min then placed on ice. The solution was then neutralized by adding 3 µl 1 M Tris (pH 8.0) and centrifuged at 12,000×*g*, 5 min. The genomic DNA containing supernatant was then transferred to a new tube and stored at −70 °C. Indels were quantitated using the Guide-it^TM^ Mutation Detection Kit (Takara Bio USA, Inc.) according to manufacturer’s protocol. The PCR products were denatured and reannealed by thermocycler and subjected to resolvase digestion (Takara Bio USA, Inc.). Digests were then subjected to agarose gel electrophoresis. The splice-blocking morpholino *rab8*-SP1 was previously described^41^. For in vivo membrane tubules imaging, *Tg(centrin:GFP)* embryos were co-injected with 125 pg of tdTom-EHD1 and ARL13B-GFP mRNA at the one cell stage.

### Expression analysis in human and zebrafish embryo

Preparation of cell and zebrafish embryo lysates were carried out as described previously^8^. Briefly, human cells and zebrafish embryos were homogenized in lysis buffer (20 mM Tris at pH 8, 137 mM NaCl, 10% glycerol, 1% Triton X-100) containing protease inhibitor cocktail (Roche). Lysates were centrifuged for 10 min at 13,000 r.p.m. Sample buffer was added to the supernatants and samples were boiled. Note the use of two different Pacsin2 antibodies for zebrafish western blot analyses. The anti-Pacsin2 antibody from Proteintech was used for *pacs1/2*CRISPR western blot in Fig. 1f. Uncropped images of immunoblots are in Supplementary Figure 8.

### Statistics and reproducibility

Statistical analyses were performed using GraphPad Prism 6 for Macintosh OS. Data presented are as specified in the figure legends or text but generally ±SEM or SD. Two-group comparisons were carried out using an unpaired, two-tailed Student *t*-test with significant values indicated on graphs in figures as follows: *P < 0.05, **P < 0.001, ***P < 0.0001, n.s. not significant, *N* number of independent experiments.

### Reporting summary

Further information on experimental design is available in the Nature Research Reporting Summary linked to this article.

## Supplementary information

Supplementary Information

Description of Additional Supplementary Files

Supplementary Movie 1

Supplementary Movie 2

Supplementary Movie 3

Supplementary Movie 4

Supplementary Movie 5

Supplementary Movie 6

Reporting Summary

## Data Availability

The FIB-SEM imaging data that support findings of this study are available in the National Cancer Institute Center for Strategic Scientific Initiatives Data Coordinating Center (https://cssi-dcc.nci.nih.gov/cssiportal/view/5c0ae7ae34b81e5d353b3607). Other data that support the findings of this study are available within the article and its Supplementary Information files or from the corresponding author upon request. A reporting summary for this article is available as a Supplementary Information file.
