# Publisher Correction: Investigation of F-BAR domain PACSIN proteins uncovers membrane tubulation function in cilia assembly and transport

**DOI:** 10.1038/s41467-019-08872-0

**Published:** 2019-02-19

**Authors:** Christine Insinna, Quanlong Lu, Isabella Teixeira, Adam Harned, Elizabeth M. Semler, Jim Stauffer, Valentin Magidson, Ajit Tiwari, Anne K. Kenworthy, Kedar Narayan, Christopher J. Westlake

**Affiliations:** 1 Laboratory of Cellular and Developmental Signaling, Center for Cancer Research, National Cancer Institute, National Institutes of Health, Frederick, MD 21702 USA; 2 Center for Molecular Microscopy, Center for Cancer Research, National Cancer Institute, National Institutes of Health, Frederick, MD 21701 USA; 3 Cancer Research Technology Program, Frederick National Laboratory for Cancer Research, Frederick, MD 21702 USA; 4 Department of Molecular Physiology and Biophysics, Vanderbilt University School of Medicine, Nashville, TN 37232 USA

Correction to: *Nature Communications*; 10.1038/s41467-018-08192-9; published online 25 January 2019.

In the original version of this Article, the fifth sentence of the abstract incorrectly read ‘Remarkably, we show that PACSIN1 and EHD1 assemble membrane t7ubules from the developing intracellular cilium that attach to the plasma membrane, creating an extracellular membrane channel (EMC) to the outside of the cell.’, and should have read ‘Remarkably, we show that PACSIN1 and EHD1 assemble membrane tubules from the developing intracellular cilium that attach to the plasma membrane, creating an extracellular membrane channel (EMC) to the outside of the cell.’. This has been corrected in both the PDF and HTML versions of the Article.

