# Supplementary material for: Investigation of F-BAR domain PACSIN proteins uncovers membrane tubulation function in cilia assembly and transport
Source: Nat Commun. 2019 Jan 25;10:428. doi: 10.1038/s41467-018-08192-9 (PMC6347608; doi:10.1038/s41467-018-08192-9)
Supplement: Supplementary file 1 — Supplementary Information [file 41467_2018_8192_MOESM1_ESM.pdf]

Investigation of F-BAR domain PACSIN proteins uncovers membrane tubulation function in cilia assembly and transport

Insinna et al.

Supplementary Information

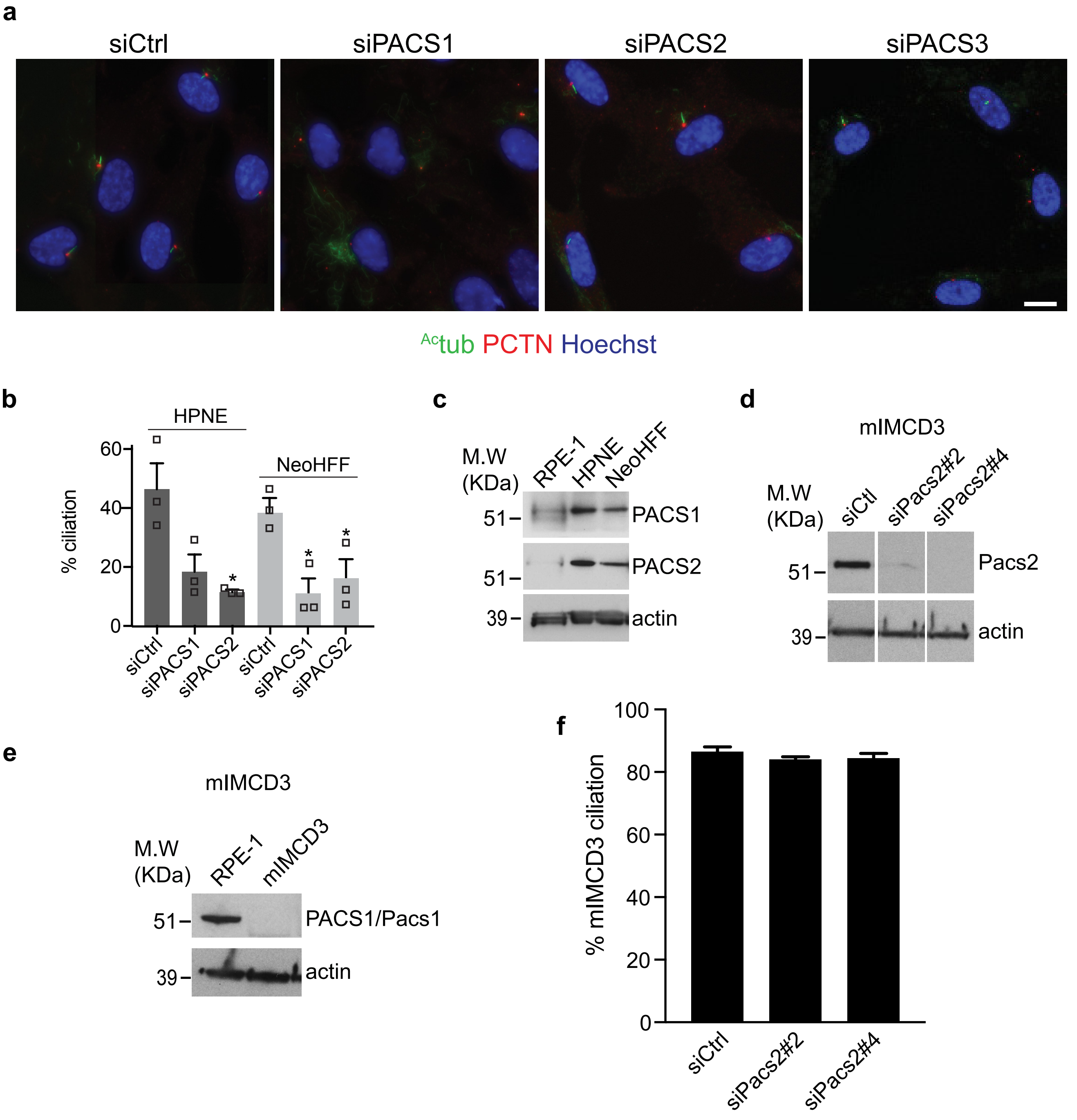

Supplementary Figure 1. PACSIN1 and PACSIN2, but not PACSIN3 function in ciliogenesis. (**a**) Epifluorescence images of cells quantified for ciliation in Fig. 1**b**. Scale bar: 10 µm. (**b**) Quantification of ciliation in HPNE (n siCtrl= 250, n siPACS1= 194, n siPACS2= 192) and NeoHFF (n siCtrl= 252, n siPACS1= 138, nsiPACS2= 154) cells treated as in **a**. Means ± S.E.M, N=3. Two-tailed t-test analysis compared with siCtrl. (**c**) Western analysis of PACSIN1 and PACSIN2 protein expression in RPE-1, HPNE, and NeoHFF cells. (**d**) Western analysis of Pacsin2 expression in mIMCD3 cells treated for 72 h with siCtrl or siPacs2#2 or siPacs2#4 (sequences shown in Supplementary Table 1). (**e**) Western analysis of PACSIN1 expression in RPE-1 and mIMCD3 cells. (**f**) Quantification of ciliation in mIMCD3 cells (n siCtrl= 502; n siPacs2#2= 581; n siPacs2#4=576), with 24 h serum starvation, followed by staining as in Fig. 1**b**. Means ± S.E.M, N=3.

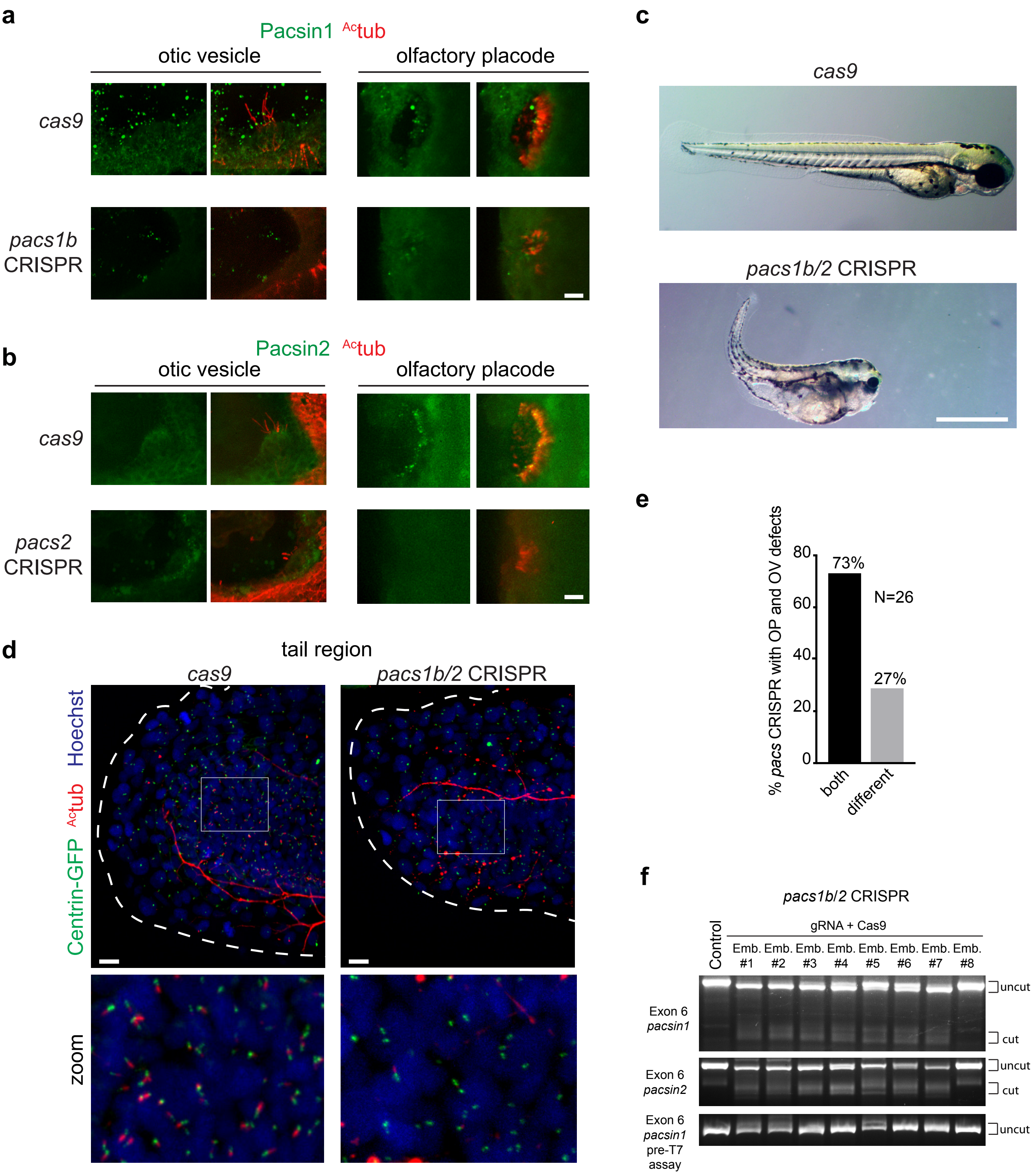

Supplementary Figure 2. Pascin expression and CRISPR knockdowns in zebrafish embryos. (**a**, **b**) Images of otic vesicles and olfactory placodes in Cas9-injected, *pacsin1b* (**a**) or *pacsin2* (**b**) CRISPR embryos at 3 dpf stained with Actub and Pacsin1 or Pacsin2 antibodies. Images were taken using identical settings to compare protein expression levels in Cas9-injected versus mutant tissues. Gene targeting specificity of CRISPR treatments was confirmed by observed reductions in fluorescence intensity for Pacsin proteins. Scale bars: 10 μm. (**c**) Brightfield images of 3 dpf zebrafish embryos injected as described in Fig. 1**f**-**j**. Note the presence of body curvature, small eye, and hydrocephalus phenotypes. Scale bar: 1 mm. (**d**) Images of ciliated cells from tails of 24hpf Tg(Cen-

trin:GFP) embryos injected with CRISRP pacs1b/2 gRNA+ Cas9 RNA or Cas9 RNA only and stained with Actub and Hoechst. Scale bar: 10µm. (**e**) Quantification of phenotypical mosaicism in 3 dpf pacsin CRISPR embryos from Fig. 1**f-j**. Note that 73% of embryos presented both OV and OP defects simultaneously (n=26 embryos). Only embryos with both organs visible were quantified in this experiment. (**f**) At 3dpf, control (lane1) and 8 randomly selected *pacs1b/2* gRNA injected F0 embryos (lane 2-10) were subjected to T7EI assay. The T7EI fragments are indicated as "cut" and wild type are noted as "uncut' . Note that the majority of pacsin gRNA injected embryos harbored indels and presented a reduction in wildtype fragments suggesting a high mutagenesis rate at the pacsin targets.

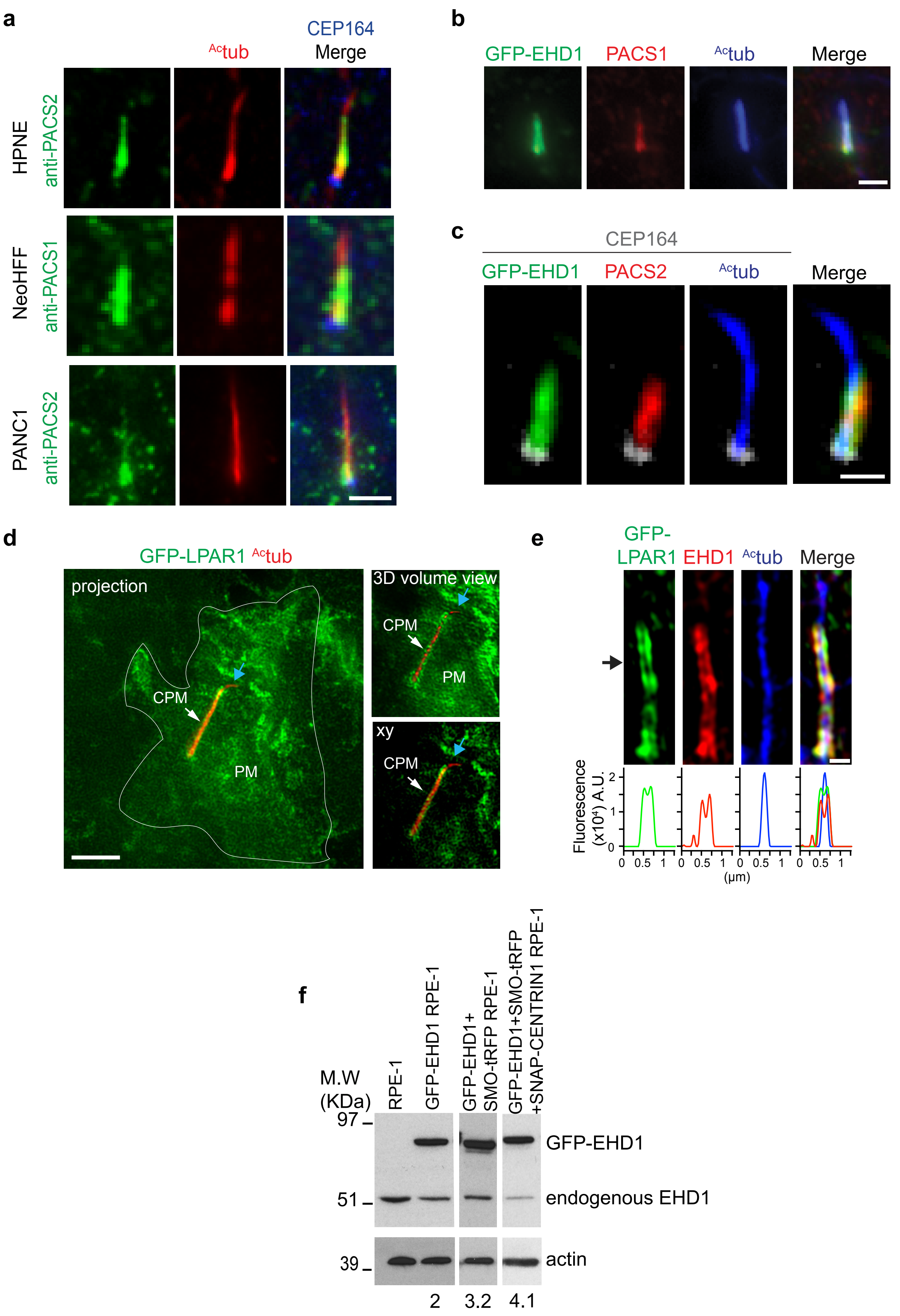

Supplementary Figure 3. Analysis of endogenous and ectopically expressed CPM proteins.

(**a**) Images of HPNE, NeoHFF, and PANC1 cells treated as in Fig. 2**a** and stained with antibodies for PACSINs, Actub and CEP164. Scale bar: 2µm. (**b**) Epifluorescence images of GFP-EHD1 cells, serum starved for 24 h, and stained with antibodies for PACSIN1 and Actub. Images are maximum projections of a z-stack. Scale bar: 2µm. (**c**) Images of GFP-EHD1 cells as in **b** stained with antibodies for PACSIN2, CEP164, and Actub. Images are maximum intensity projections of deconvolved z-stacks. Scale bar: 500 nm. (**d**) Images taken with Zeiss SIM microscope of RPE-1 cells transiently transfected with GFP-LPAR1, serum starved for 24 h, and stained with Actub antibody. Images are maximum intensity projections of z-stacks, 3D volume view (generated using Slidebook software), or single xy planes as indicated. Cell outline (white) in left image shows PM GFP-LPAR1. White arrows indicate CPM, blue arrow shows the distal cilia tip region outside of the CPM and cell. Scale bar: 2µm. (**e**) Images (top panels) of GFP-LPAR1 cells treated as in **d**, and stained with EHD1 and Actub antibody. Images are single xy planes taken with 63x objective. Bottom panels show fluorescence intensity profile plots corresponding to the direction of the black arrow shown in top panels. Scale bar: 500 nm. (**f**) Western blot analysis comparing GFP-EHD1 over-expression levels in GFP-EHD1, GFP-EHD1 + SMO-tRFP cells and the triple line. Comparison of GFP-EHD1 to endogenous EHD1 shows that the GFP-fusion protein is expressed 2, 3.2 and 4.1 fold higher in the indicated cells line.

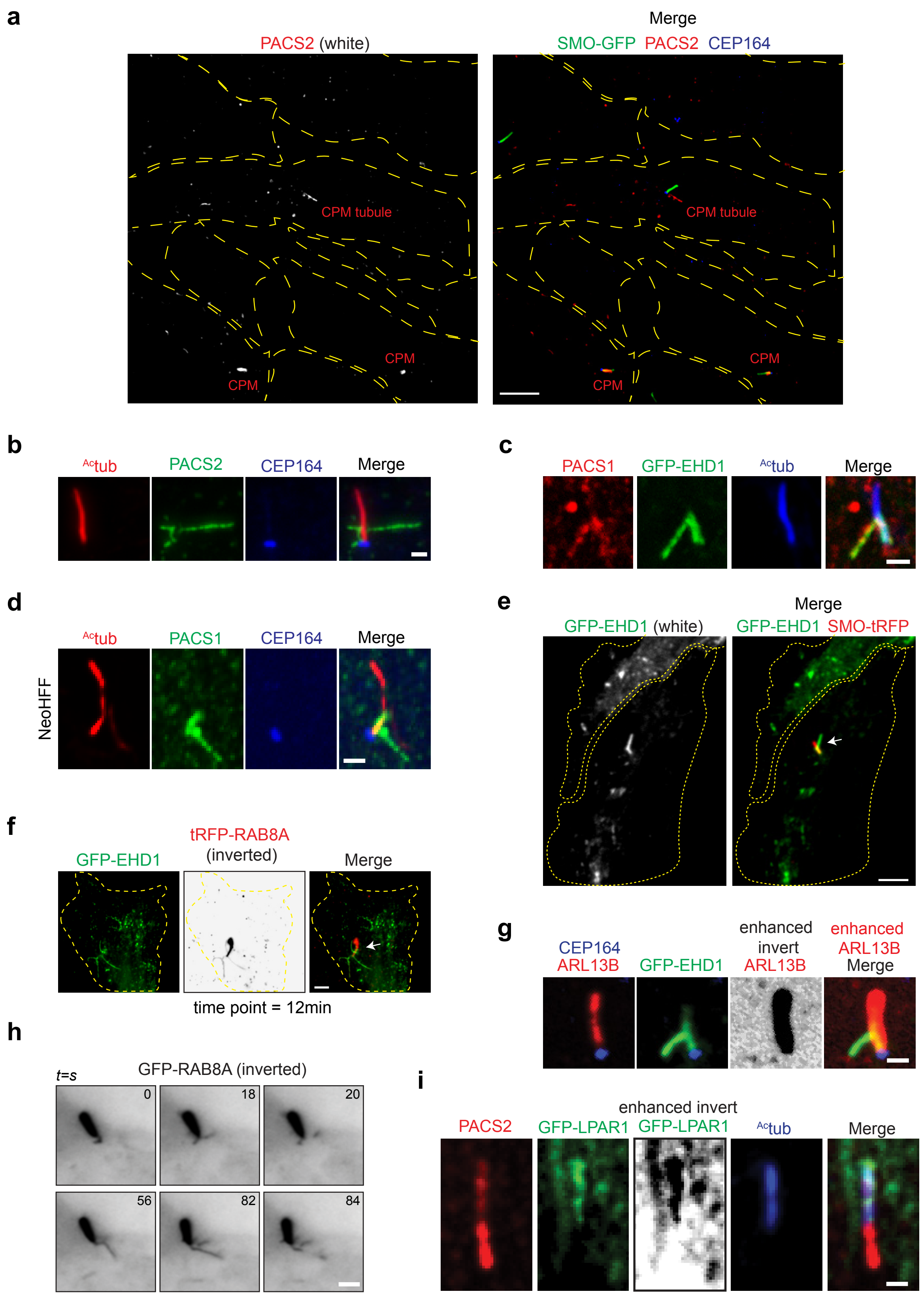

Supplementary Figure 4: Examination of protein localization in CPM tubules. (**a**) Epifluorescence image of GFP-SMO cells stained with antibodies for PACSIN2 and CEP164. Yellow dotted lines show the cell outline. (**b**) Images of RPE-1 cells stained with antibodies for PACSIN2, CEP164

and Actub and imaged as in **a**. (**c**) Images of GFP-EHD1 cells stained with antibodies for PACSIN1 and Actub and captured using a CMOS camera. (**d**) Images of NeoHFF cells stained with antibodies for PACSIN1, CEP164, and Actub. Nearest-neighbor deconvolution was applied. (**e**) Whole cell view of live image (t= 4 min) in Fig. 3**g** with yellow dotted lines showing cell outline and white arrow indicating a CPM tubule. (**f**) Whole cell view of live image (t= 12min) in Fig. 3**h** as in **e**. (**g**) Representative images of GFP-EHD1 cells stained with antibodies for ARL13B and CEP164. Middle right panel shows a contrast-enhanced, inverted image of the red ARL13B channel to demonstrate the absence of ARL13B signal in tubules. Merge channel also shows an enhanced version of the red channel. (**h**) Epifluorescence time-lapse imaging of GFP-RAB8A cells. GFP-RAB8A is observed in the ciliary membrane and on dynamic tubules at the base of the cilium. Membrane tubules form near the base of the cilium and subsequently fragment and disperse into the cytoplasm. RAB8A signal was inverted to enhance the visualization of membrane tubules (n= 25 cells). (**i**) Images of RPE-1 cells transiently expressing GFP-LPAR1 and stained with antibodies for PACSIN2 and Actub. Both GFP-LPAR1 and PACSIN2 are found in the CPM. Middle panel shows a contrast-enhanced, inverted image of the green GFP-LPAR1 channel to demonstrate the absence of LPAR1 in membrane tubules. Cells in **a**-**i** were serum starved for 24 h. **c**, **d**, **f** and **i**, were imaged with a 100x objective, other images were captured with 63x objective. Images shown in **b**-**d**, **g**-**i** are single xy planes and all other images are maximum projections. Scale bars: 1 µm except for **a**, **e** and **f**; scale bar: 5 µm.

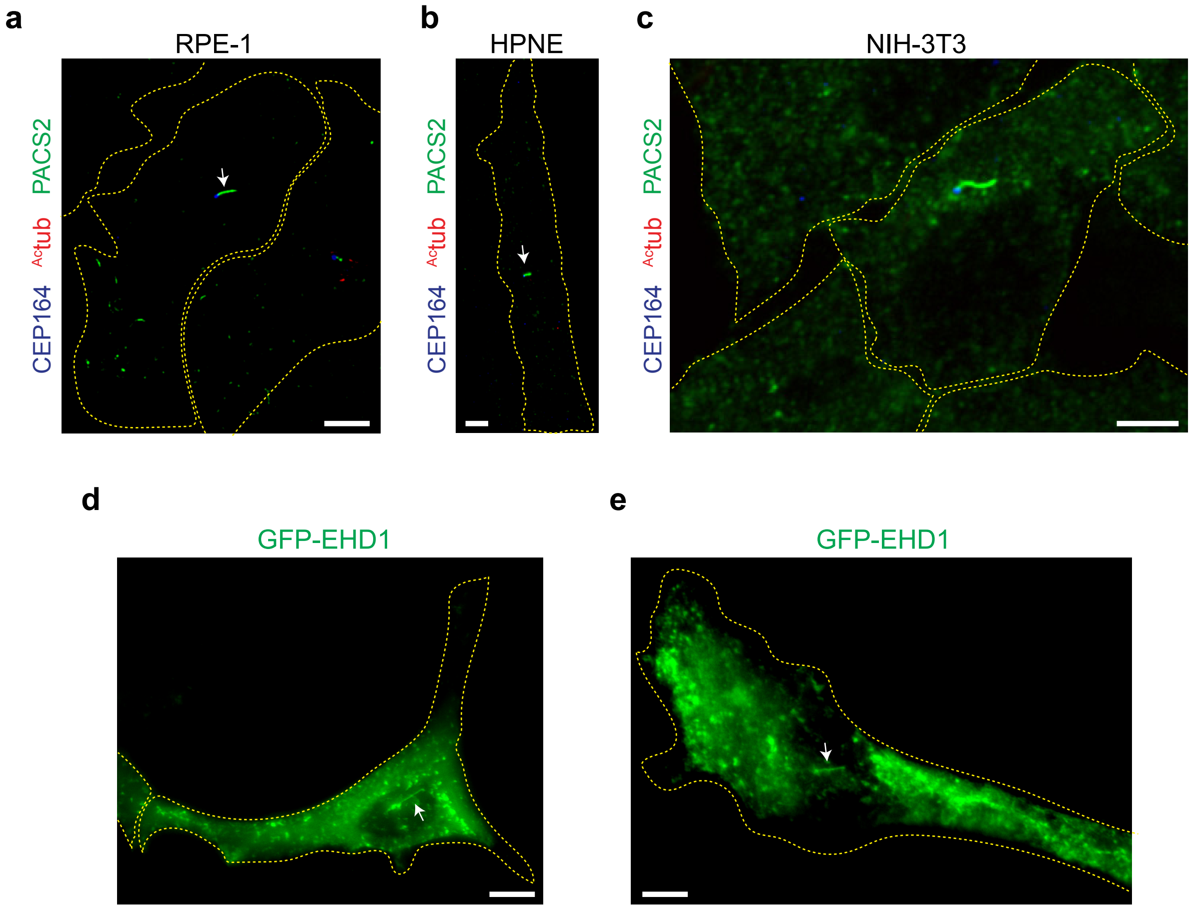

Supplementary Figure 5: Whole cell views of different cell lines with EMCs. (**a**) RPE-1 cell from Fig. 6**c**, (**b**) HPNE cell from Fig. 6**g**, (**c**) NIH-3T3 cell from Fig. 6**h**, and (**d**, **e**) GFP-EHD1 cells from Fig. 7**a**, **b** with yellow dotted lines showing the outlines of cell membranes. White arrow indicates position of MC-associated tubules. Scale bar: 5 μm

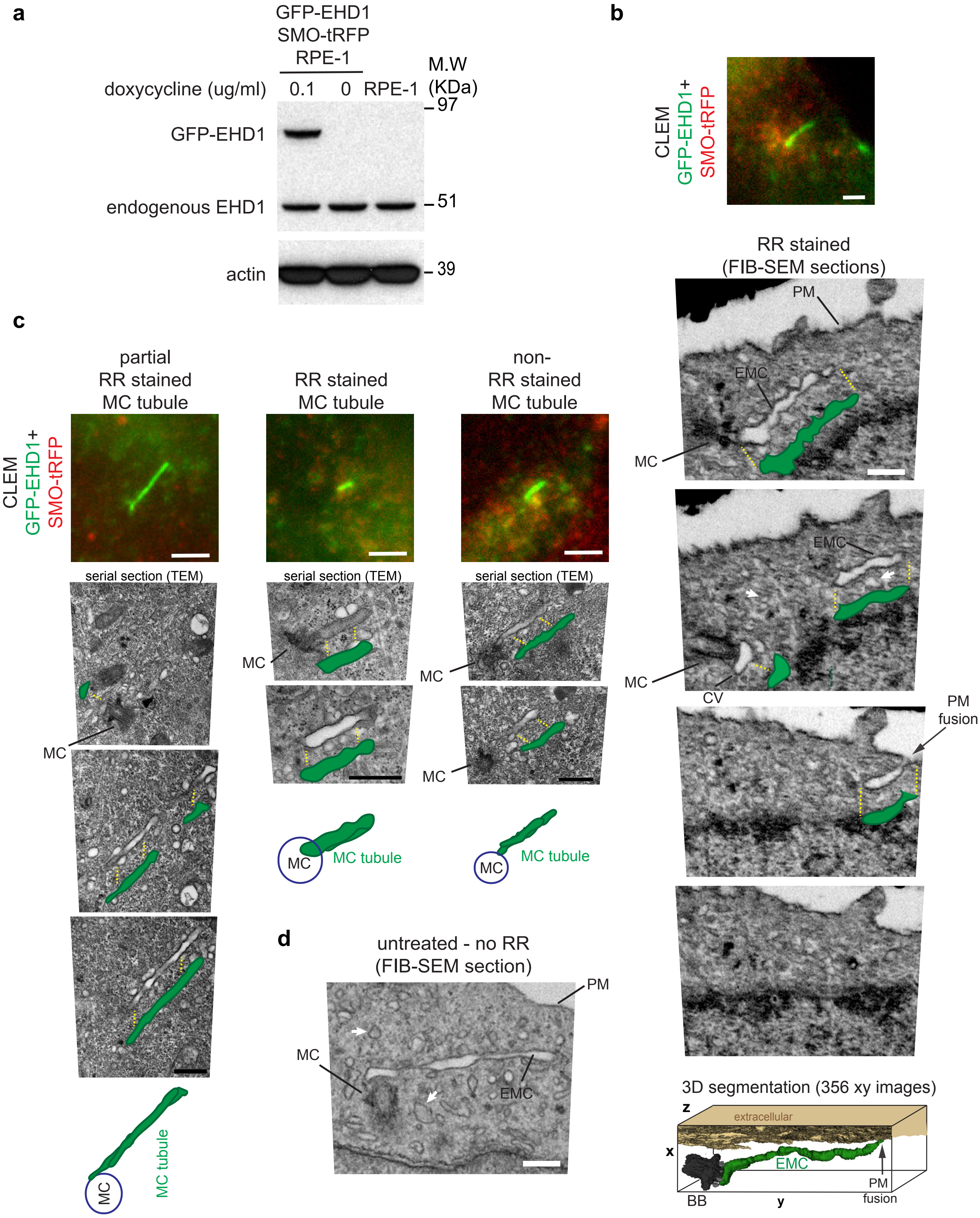

a
GFP-EHD1
SMO-tRFP
RPE-1
doxycycline (ug/ml)
0.1
0
RPE-1
M.W
(KDa)
97
GFP-EHD1
endogenous EHD1
51
actin
39
b
CLEM
GFP-EHD1+
SMO-tRFP
RR stained
(FIB-SEM sections)
PM
EMC
MC
CV
PM
fusion
3D segmentation (356 xy images)
extracellular
EMC
BB
PM
fusion
c
partial
RR stained
MC tubule
non-
serial section (TEM)
MC tubule
d
untreated - no RR
(FIB-SEM section)

Supplementary Figure 6: Ruthenium red (RR) staining of MC-associated membrane tubules. (**a**) Western blot analysis showing GFP-EHD1 expression levels in GFP-EHD1 + SMO-tRFP cells used in **b** and **c** and incubated with 0.1 μg per ml of doxycycline for 24h resulting in GFP-EHD1 expression comparable to endogenous EHD1. (**b**) Top panel shows epifluorescence images of a serum starved GFP-EHD1 + SMO-tRFP cell treated as in **a** used for CLEM/FIB-SEM. Scale bar: 2 μm. Middle panels show cropped FIB-SEM images with the mother centriole (MC) and associated membrane tubule stained with RR. EMC is highlighted in green and shifted. Yellow dotted lines show alignment of the EMC and highlighted tubule. Scale bar: 500 nm. Bottom panel shows the 3D segmentation analysis of the FIB-SEM images. BB (black), CPM and EMC tubules (green). The position of EMC/PM fusion is indicated with black arrows. White arrows indicate intracellular membrane structures. (**c**) Top panels show CLEM epifluorescence images of serum starved GFP-EHD1 + SMO-tRFP cells as in **b**, with MC-membrane tubules (top panels). Scale bar: 2 μm. Middle panels show TEM images of serial sections of the region of the cell shown in the top panels. Images from 9 cells with detectable MC- tubules; 3 cells showed RR stained MC- tubules (RR stained MC tubule), 3 cells showed darker RR staining nearer the PM end of the tubules (partial RR stained MC tubule), and 3 cells did not show RR staining (non-RR stained MC-tubule). RR staining determinations were made by comparing the intensity of the MC-tubules with other intracellular membranes. Scale bar: 500 nm. Bottom panel represents the merged ciliary structure traces from the serial sections. (**d**) FIB-SEM image of the cell in Fig. 7**a** showing a section of an EMC that has not been treated with RR. The EMC and surrounding intracellular membranes have similar intensity staining/contrast. White arrows indicate intracellular membrane structures. Scale bar: 500 nm.

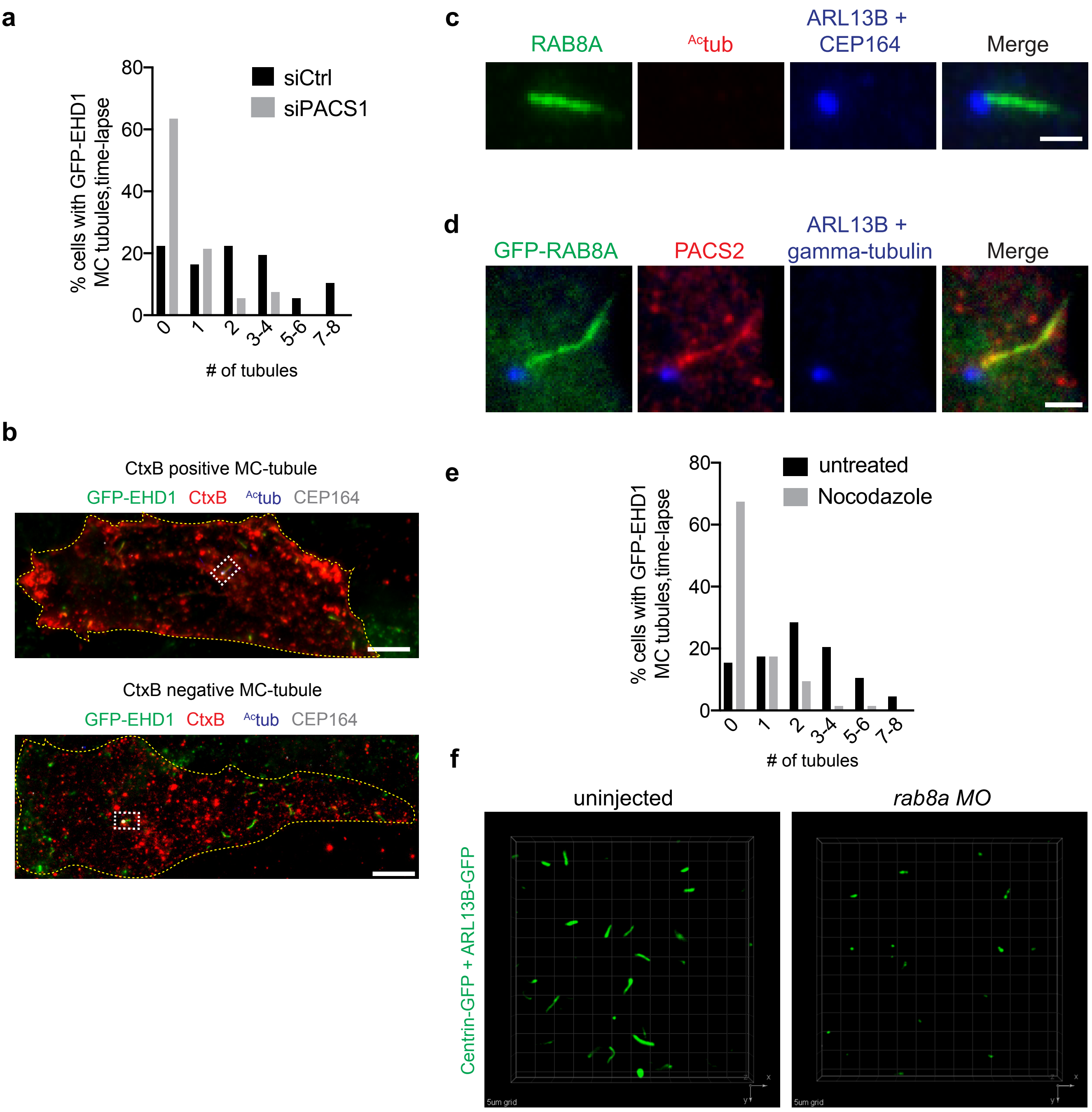

Supplementary Figure 7: The EMC is PACSIN1 and microtubule dependent and contains RAB8. (**a**) Quantification of GFP-EHD1 tubules frequency in the triple line treated with siPACS1 from Fig. 8**b**. Graph shows the percentage of cells (y axis) with indicated MC-associated tubule number (x axis). (**b**) Whole cell view of GFP-EHD1 cells labeled with CtxB as shown in Fig.8**f**. Images are maximum intensity projections taken with SDC microscope (white box marks the MC area of the cell). Scale bar: 5 µm. (**c**) Epifluorescence images of RPE-1 cells serum starved for 3 h and stained with RAB8A, ARL13B, Actub and CEP164 antibodies. (**d**) Epifluorescence images of GFP-RAB8A cells serum starved for 3 h and stained with PACSIN2, ARL13B, and gamma-tubulin antibodies. Images in **c**, **d** were taken with 63x objective. Scale bars: 2µm. (**e**) Quantification of

GFP-EHD1 tubules frequency in triple line treated with Nocodazole from Fig. 8**h**. (**f**) Representative images of cilia in the tail region of a 24hpf Tg(Centrin:GFP) zebrafish embryo injected with ARL13B-GFP and rab8 MO. Live images are 3D volume views taken with 63x objective and CMOS camera. Grid size 5μm.

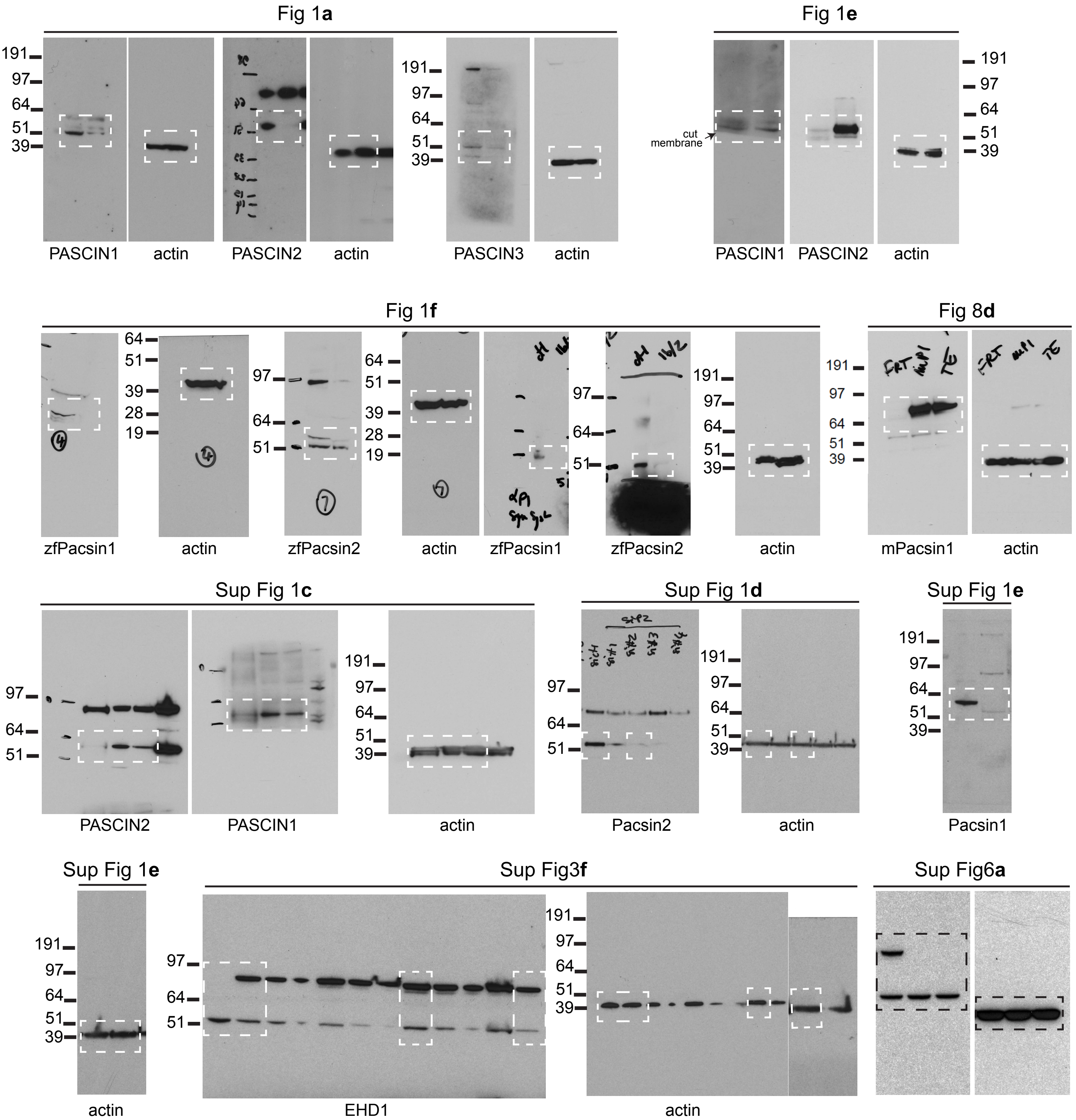

Supplementary Figure 8: Full scans of western blots.

Table S1 -

Gene	siRNA sequence
hPACSIN1	siPACSIN1 - UGACAGAGGCAGACAAGGU
hPACSIN2	siPACSIN2- CAAAUUAUGUGGAGGCGAU
hPACSIN3	siPACSIN3- GGACAUGGAACAGGCCUUU
mPacsin2	siPacsin2#2- CAAAGCAGCAGAUGCGGUA<br>siPacsin2#4- GAAGAAGGCUGUUGACGGU
**Gene**	**gRNA sequence**
zfpacsin1b	Exon2-5'-3'TTA ATA CGA CTC ACT ATA GGA CCA CAG ACA GCT TCT GGG GTT TTA GAG CTA GAA ATA<br>Exon3-5'-3'TTA ATA CGA CTC ACT ATA GGC GCA CAG TGA AGC GCA TTG GTT TTA GAG CTA GAA ATA
zfpacsin2	Exon2-5'-3'TTA ATA CGA CTC ACT ATA GGT CCA GCG ACA GCT TCT GGG AGG GTT TTA GAG CTA GAA
zfpacsin1b+2	Pan-5'-3'TTA ATA CGA CTC ACT ATA GGC CGC AGT ACA TGG AGA ACA GTT TTA GAG CTA GAA ATA
	**PCR sequence**
pac1b_Ex6_F	5'-TAA AAT GCT GTT TGC TTC GTG T-3'
pac1b_Ex6_R	5'-GAT CAG ATG ACT GAT TTG CAG C-3'
pac2_Ex6_F	5'-GTG TGT TAC AGA CAA AGG AGC G-3'
pac2_Ex6_F	5'-ACG GTG GTG TAC CTG AGA GAA T-3'

Supplementary Table 1: siRNA, CRISPR and PCR sequences.

Table S2 -

Cell Line	Ciliary structures with attached tubules	CPM with attached tubules	intracellular cilia/CV with attached tubules	Extracellular membrane channel (EMC)
GFP-EHD1+SMO-tRFP RPE-1 (3h starve, CLEM)				
1ug/ml doxycycline (FIB-SEM)	7/7 (100%)	2/7 (29%)	5/7 (71%)	3/5 (60%)
0.1ug/ml doxycycline + RR (FIB-SEM)	3/4 (75%)	0/4* (0%)	3/4 (75%)	2/3 (66.6%)
0.1ug/ml doxycycline + RR (serial section TEM)	9/9 (100%)	0/9 (0%)	9/9 (100%)	n/a
GFP-CENTRIN1 RPE-1 (3h starve, CLEM)	3/19 (16%)	0/0 (0%)	3/3 (100%)	1/3 (33.3%)

*cell with a cilium attached to PM and a GFP-EHD1 tubule adjacent to the cilium

Supplementary Table 2: Ciliary structures detected by FIB-SEM and serial section TEM. Ciliary structures with attached tubules detected in GFP-EHD1 and GFP-CENTRIN1 cells. Data column includes DAV, CV, intracellular cilia, and mature cilia (fused with PM) stages detected after 3h starvation. Doxycyclin treatments were for 24h. CLEM – correlative light and electron microscopy, MC – mother centriole, CV - ciliary vesicle, EMC – extracellular membrane channel, CPM – ciliary pocket membrane.
