# Supplementary material for: Investigation of F-BAR domain PACSIN proteins uncovers membrane tubulation function in cilia assembly and transport
Source: Nat Commun. 2019 Jan 25;10:428. doi: 10.1038/s41467-018-08192-9 (PMC6347608; doi:10.1038/s41467-018-08192-9)
Supplement: Supplementary file 2 — Description of Additional Supplementary Files [file 41467_2018_8192_MOESM2_ESM.pdf]

Description of Additional Supplementary Files

File Name: Supplementary Movie 1
Description: GFP-EHD1 and tRFP-RAB8 co-localize on CPM tubules. Time-lapse image series of a GFP-EHD1 RPE-1 cell transiently expressing tRFP-RAB8A as shown in Fig. 3h. TIRF images were taken every minute for 15 min after 24 h starvation.

File Name: Supplementary Movie 2
Description: FIB-SEM of CPM tubule. Raw *XY* FIB-SEM image series from GFP-EHD1 + SMO-tRFP RPE-1 cells following 3 h starvation as shown in Fig. 4. Black arrows indicate the two CPM tubules and CPM-PM fusion, white arrows follow the cilium. Movie were created with Wondershare Filmora and iMovie. CPM, ciliary pocket membrane; PM, plasma membrane; BB, basal body.

File Name: Supplementary Movie 3
Description: FIB-SEM of EMC connecting a CV and the PM. Raw *XY* FIB-SEM image series of a GFP-EHD1 + SMO-tRFP RPE-1 cell following 3 h starvation as shown in Fig. 7 **a**. In the raw FIB-SEM images, a region of the EMC is outlined to better show the connection to the PM. Black arrows indicate the CV and the EMC, the white arrow shows the PM fusion site. Movie was generated as in movie 2. CV, ciliary vesicle; EMC, extracellular membrane channel; BB, basal body.

File Name: Supplementary Movie 4
Description: FIB-SEM of EMC connecting a short intracellular cilium and the PM. Raw *XY* FIB-SEM image series as shown in Fig. 7 **b** and as described in Supplementary Movie 3. Structures are indicated by white arrows.

File Name: Supplementary Movie 5
Description: FIB-SEM of an EMC connecting the CV and PM in GFP-CENTRIN1 cells. Raw *XY* FIB-SEM image series of a GFP-CENTRIN1 RPE-1 cell following 3 h starvation as shown in Fig. 7**c**. Black arrows indicate the CPM tubule. Movies were generated using image J and iMovie.

File Name: Supplementary Movie 6
Description: Early ciliary membrane tubules are dynamic and form from the developing intracellular cilium. Time-lapse video of a RPE-1 cell expressing GFP-EHD1, SMO-tRFP, and SNAP-CENTRIN1 (SNAP-647-treated) and starved for 3h as shown in Fig. 8**e**. SDC image z-stacks were taken every 1 min. Images shown are a single xy plane.
