# Supplementary material for: Investigation of F-BAR domain PACSIN proteins uncovers membrane tubulation function in cilia assembly and transport
Source: Nat Commun. 2019 Jan 25;10:428. doi: 10.1038/s41467-018-08192-9 (PMC6347608; doi:10.1038/s41467-018-08192-9)
Supplement: Supplementary file 9 — Reporting Summary [file 41467_2018_8192_MOESM9_ESM.pdf]

nature research

Corresponding author(s): Christopher J. Westlake

Reporting Summary

Nature Research wishes to improve the reproducibility of the work that we publish. This form provides structure for consistency and transparency in reporting. For further information on Nature Research policies, see Authors & Referees and the Editorial Policy Checklist.

Statistical parameters

When statistical analyses are reported, confirm that the following items are present in the relevant location (e.g. figure legend, table legend, main text, or Methods section).

n/a	Confirmed	
☐	☒	The exact sample size (*n*) for each experimental group/condition, given as a discrete number and unit of measurement
☐	☒	An indication of whether measurements were taken from distinct samples or whether the same sample was measured repeatedly
☐	☒	The statistical test(s) used AND whether they are one- or two-sided<br>*Only common tests should be described solely by name; describe more complex techniques in the Methods section.*
☒	☐	A description of all covariates tested
☒	☐	A description of any assumptions or corrections, such as tests of normality and adjustment for multiple comparisons
☐	☒	A full description of the statistics including central tendency (e.g. means) or other basic estimates (e.g. regression coefficient) AND variation (e.g. standard deviation) or associated estimates of uncertainty (e.g. confidence intervals)
☒	☐	For null hypothesis testing, the test statistic (e.g. *F*, *t*, *r*) with confidence intervals, effect sizes, degrees of freedom and *P* value noted<br>*Give P values as exact values whenever suitable.*
☒	☐	For Bayesian analysis, information on the choice of priors and Markov chain Monte Carlo settings
☒	☐	For hierarchical and complex designs, identification of the appropriate level for tests and full reporting of outcomes
☒	☐	Estimates of effect sizes (e.g. Cohen's *d*, Pearson's *r*), indicating how they were calculated
☐	☒	Clearly defined error bars<br>*State explicitly what error bars represent (e.g. SD, SE, CI)*

Our web collection on statistics for biologists may be useful.

Software and code

Policy information about availability of computer code

Data collection	No computer code was used to collect the data.
Data analysis	Graph Pad Prism 6 for Macintosh OS

For manuscripts utilizing custom algorithms or software that are central to the research but not yet described in published literature, software must be made available to editors/reviewers upon request. We strongly encourage code deposition in a community repository (e.g. GitHub). See the Nature Research guidelines for submitting code & software for further information.

Data

Policy information about availability of data

All manuscripts must include a data availability statement. This statement should provide the following information, where applicable:

- Accession codes, unique identifiers, or web links for publicly available datasets
- A list of figures that have associated raw data
- A description of any restrictions on data availability

No unique material was used in this study. A list of commonly used materials is described in the method section of the manuscript.

Field-specific reporting

Please select the best fit for your research. If you are not sure, read the appropriate sections before making your selection.

☒ Life sciences ☐ Behavioural & social sciences ☐ Ecological, evolutionary & environmental sciences

For a reference copy of the document with all sections, see nature.com/authors/policies/ReportingSummary-flat.pdf

Life sciences study design

All studies must disclose on these points even when the disclosure is negative.

Sample size	Ciliation and ciliary structure counts are consistent with quantifications in our prior publication (Lu et al, NCB 2015) and other cilia papers.<br>TEM counts - are higher than typically reported by other publications on ciliogenesis<br>FIB-SEM counts are higher than reported by other publications for this technique<br>Zebrafish organs defects and cilia counts are consistent with quantifications in our prior publication (Lu et al, NCB 2015) and other cilia papers.
Data exclusions	No data were excluded from our analyses.
Replication	All attempts at replications were successful.
Randomization	For mammalian cell work and zebrafish experiments, each treatment condition was assigned a random number, which was not revealed to the investigator until final outcome assessment. For CLEM-FIBSEM/TEM analyses, sample identity was known during the entire experiment.
Blinding	The investigators were blinded to allocation during experiments but not during outcome assessment. For CLEM-FIBSEM/TEM data analyses, the investigators were not blinded.

Reporting for specific materials, systems and methods

Materials & experimental systems

n/a	Involved in the study
☒	☐ Unique biological materials
☐	☒ Antibodies
☐	☒ Eukaryotic cell lines
☒	☐ Palaeontology
☐	☒ Animals and other organisms
☒	☐ Human research participants

Methods

n/a	Involved in the study
☒	☐ ChIP-seq
☒	☐ Flow cytometry
☒	☐ MRI-based neuroimaging

Antibodies

Antibodies used	Commercial antibodies used were as follows: anti-Acetylated tubulin (Actub, clone 6-11B-1, 1/10000, Sigma), anti-Gamma-tubulin (GTU-88, 1/1000, Sigma), anti-b-actin (clone AC-15, 1/30000, Sigma), anti-PACSIN1 (1/100, Synaptic Systems), anti-PACSIN2 (1/250, Abcam), anti-PACSIN2 (1/500, Proteintech), anti-PACSIN3 (1/100, Abcam), anti-Pericentrin (PCTN, 1/5000, Novus Biologicals), anti-EHD1 (1/500, Novus Biologicals), anti-RPGRIP1L (1/200, Proteintech), anti-TMEM67 (1/200, Proteintech), anti-CEP164 (1/500, Santa Cruz), anti-CP110 (1/1000, Proteintech), anti-CEP97 (1/1000, Bethyl), anti-Arl13b (1/300, clone N295B/66, NeuroMab), anti-GFP Alexa 568 and Alexa 488 (1/1000, Molecular Probes Life Technologies), Phalloidin conjugated with Alexa 488 (1/50, Molecular Probes Life Technologies), Hoechst (1/3000, Molecular Probes Life Technologies) and all secondary antibodies were from Life Technologies. The rabbit anti-RAB8A antibody was a gift from Johan Peränen (University Helsinki, Finland).
Validation	All commercial antibodies were validated by manufacturer and anti-Rab8a antibody was previously described in publications.

Eukaryotic cell lines

Policy information about cell lines

Cell line source(s)	hTERT RPE-1 , PANC1 , hTERT HPNE , NIH3T3 , HFF-1 , and IMCD3 were originally obtained from ATCC.
Authentication	Authentication was provided by ATCC.

Mycoplasma contamination	All cell lines tested negative for mycoplasma contamination.
Commonly misidentified lines (See ICLAC register)	No commonly misidentified cell lines were used.

Animals and other organisms

Policy information about studies involving animals; ARRIVE guidelines recommended for reporting animal research

Laboratory animals	Zebrafish animals used in this study were wildtype TAB-5.
Wild animals	The study did not involve wild-type animals.
Field-collected samples	The study did not involve samples collected from the field.
